# Site-Selective Modification of Lanthanum Oxychloride to Modulate Halide-Ion Conduction

**DOI:** 10.1021/acsaem.6c00392

**Published:** 2026-04-16

**Authors:** Jingxiang Cheng, Victor Alexander Gomez, Alice R. Giem, Carlos A. Larriuz, Samuel Franz Gatti, Adrian F. Silva, Lucia Zuin, Sigita Trabesinger, Sarbajit Banerjee

**Affiliations:** † Department of Chemistry, 14736 Texas A&M University, College Station, Texas 77843-3012, United States; ‡ Department of Material Science and Engineering, Texas A&M University, College Station, Texas 77843-3012, United States; § Laboratory for Inorganic Chemistry, Department of Chemistry and Applied Biosciences, ETH Zurich, Vladimir-Prelog-Weg 2, CH-8093 Zürich, Switzerland; ∥ Laboratory for Battery Science, PSI Center for Energy and Environmental Sciences, Paul Scherrer Institute, Forschungsstrasse 111, CH-5232 Villigen PSI, Switzerland; ⊥ PSI Center for Energy and Environmental Sciences, Paul Scherrer Institute, Forschungsstrasse 111, CH-5232 Villigen PSI,Switzerland; # School of Engineering, École Polytechnique Fédérale de Lausanne (EPFL), Lausanne 1015, Switzerland; 7 Department of Nuclear Engineering, Texas A&M University, College Station, Texas 77843-3012, United States; 8 Canadian Light Source, University of Saskatchewan, Saskatoon, SK S7N 2 V3, Canada

**Keywords:** chloride-ion conduction, beyond-lithium batteries, lanthanum oxychloride, aliovalent alloying, vacancies, lattice dynamics, all solid-state batteries, solid electrolytes

## Abstract

Design principles for solid-state halide-ion conduction remain poorly defined despite the increasing importance of halide ions as charge carriers in a variety of energy storage and electrochemical computing technologies. Here, we employ a site-selective modification strategy in which aliovalent cations are preferentially introduced at the La^3+^ crystallographic site of LaOCl in the 2*c* Wyckoff position, enabling controlled generation of chloride vacancies and modification of lattice dynamics to enhance chloride-ion conductivity. Aliovalent substitution of La^3+^ with Mg^2+^, Ca^2+^, and Sr^2+^ generates charge-compensating Cl vacancies while preserving the matlockite crystal structure. X-ray excited optical luminescence measurements with Dy^3+^ as a reporter chromophore evidence vacancy-derived midgap electronic states and an extended energy range of radiation-less Auger electron emission corresponding to substantial modification of electronic structure and local electrostatic potentials. Ca alloying at 8–10 at. % increases the chloride-ion conductivity by three- to 4 orders of magnitude as compared to unalloyed LaOCl, whereas comparable amounts of Sr- and Mg-alloying in LaOCl imbue less pronounced conductivity enhancements. Temperature-dependent Raman spectroscopy measurements reveal that Ca- and Sr-alloying substantially soften the La–Cl sublattice and yield a more compliant crystal lattice that can deform to accommodate Cl-ion migration. Structure solutions derived from Rietveld refinements to powder X-ray diffraction reveal larger O–La–Cl bond-angle deviations and enhanced out-of-plane cation displacements for Ca- and Sr-alloyed compositions as compared to Mg-alloyed LaOCl. Such local distortions enhance chloride-ion mobility by reshaping and flattening vacancy migration energy landscapes and by modulating lattice dynamics governing anion conduction. We further illustrate that coalloying of Ca with Mg and Sr induces a nonmonotonic conductivity–defect stoichiometry relationship that can be rationalized based on cooperative interactions. Together, these results establish site-selective aliovalent alloying of LaOCl as an effective route to halide-ion solid electrolytes and provide broadly generalizable design principles for site-selective modification to induce vacancy formation and lattice softening to engender facile anion transport.

## Graphical abstract

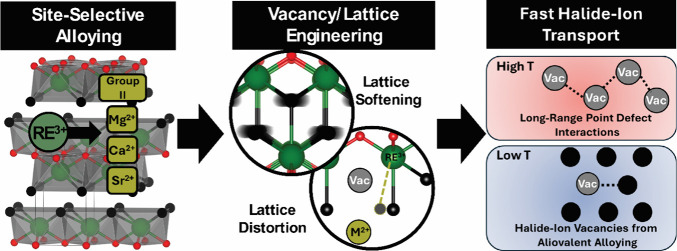

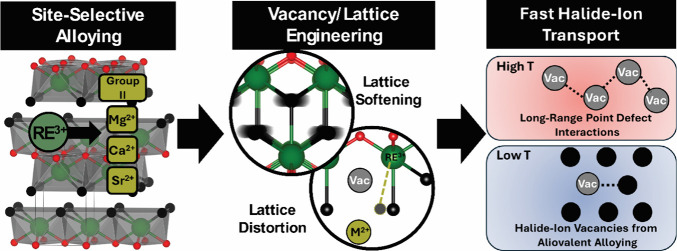

## Introduction

Solid-state ion conductors underpin a wide range of electrochemical technologies, including all-solid-state batteries, solid oxide fuel cells, electrochemical random access memory, direct lithium extraction electrodes, and ion-selective membranes.^1–3^ While proton-, lithium-, and sodium-ion conductors have received the most attention, there is increasing interest in anion conductors beyond oxygen for applications ranging from halide batteries to gas sensing and purification and corrosion inhibition.^4,5^ In these systems, achieving high anion conductivity must be balanced against demanding requirements of high-temperature operation, chemical stability, mechanical robustness, and compatibility with electrified electrode interfaces.^4–7^ Mechanisms of anion migration and concomitant lattice dynamics are underexplored and represent a key gap in the development of structure—composition—function correlations. In this Article, we explore site-selective modification of the cation sublattice of LaOCl as a means of modulating vacancy structure, lattice dynamics, and chloride-ion conduction.

Lanthanum oxychloride (LaOCl) is a chemically and thermally stable material that crystallizes in a matlockite layered crystal structure with alternating (LaO)^+^ and chloride-ion slabs. LaOCl has been extensively studied as an X-ray and optical phosphor, radiation-activated scintillator, chlorination catalyst, and solid electrolyte.^8^ The matlockite structure comprises nine-coordinated La^3+^ centers coordinated to four oxide-ions within the underlying (LaO)^+^ slab and four chloride-ions in an adjacent chloride-ion layer with a ninth longer La–Cl interaction to a distal chloride-ion in the next layer.^8–10^ The distinctive chloride-ion slabs hold promise for anisotropic vacancy hopping mechanisms. However, strategies for introducing halide vacancies and a mechanistic understanding of how specific dopants reshape the defect and migration energy landscapes and lattice dynamics governing anion conduction remain poorly understood.^11–14^

Aliovalent substitution on the La sublattice offers an attractive lever for finely modulating the concentration, local structure, and mobility of charge-compensating Cl vacancies. Substitution of La^3+^ with divalent cations introduces vacancies on the halide sublattice that can serve as mobile carriers, while simultaneously modifying the local and average structure and altering the sequence of coordination environments and their energy positioning along diffusion pathways.^7,15^ Previous work on Ca-substituted LaOCl demonstrated that chloride-ion conductivity can be enhanced by several orders of magnitude at elevated temperatures, which suggests that appropriate tuning of the substituent cation radius and charge can render the LaOCl framework more conducive to vacancy transport.^14^ However, aliovalent substitution induces diverse perturbations to the local and long-range structure impacting both transition state energetics and collective phonon dynamics with varied implications for flattening of the migration energy landscape. In this Article, we systematically probe the impact of substitution of La^3+^-ions with divalent alkaline-earth metals to explore how site-selective modification alters lattice dynamics and ion conduction.

Beyond single dopants, combining multiple alloying species introduces the possibility of cooperative or antagonistic interactions between defects.^11–13,16^ Studies on coalloyed correlated oxides have shown that functional properties can exhibit nonlinear dependences on the concentrations of two dopants, with interaction terms that either amplify or mitigate the individual dopant effects. Translating this concept to LaOCl suggests that appropriately chosen coalloying combinations could further enhance chloride-ion conductivity by simultaneously optimizing vacancy concentration, lattice compliance, and defect interactions, while compensating for local site trapping or large structural distortions. In this Article, we examine divalent Group II cations, Mg^2+^, Ca^2+^, and Sr^2+^ as individual and coalloyed aliovalent substituents to span a representative range of ionic radii while maintaining redox stability. Site-selective modification and its impact on structure and composition are examined by Rietveld refinements to powder X-ray diffraction corroborated through electron microscopy, and instrumental neutron activation analysis as well as limited neutron powder diffraction measurements. Structural information obtained from Rietveld refinements yields lattice parameters, bond angles, and cation displacements, thereby quantifying the role of aliovalent alloying in modifying the LaOCl framework. Dy^3+^ is homovalently coalloyed on the La site in low concentrations as a spectroscopic reporter to probe changes in local coordination and the balance between radiative and nonradiative decay channels using synchrotron-based soft X-ray absorption spectroscopy and X-ray-excited optical luminescence (XEOL).^17,18^ Chloride-ion transport is quantified by temperature-variant electrochemical impedance spectroscopy in the temperature range of 25–400 °C, enabling mapping of ion conduction to composition and structure. To connect transport with lattice dynamics, variable-temperature Raman spectroscopy is used to track the evolution of Cl-centered A_1g_ and E_g_ phonons, from which a Raman-based thermal expansion proxy and an effective anharmonicity parameter are extracted. These descriptors provide quantitative measures of lattice stiffness and phonon–defect scattering that can be correlated with ion conductivity. Ion conduction mechanisms are further examined using first-principles density functional theory (DFT) calculations, including vacancy formation energy analyses, nudged elastic band calculations of ion migration pathways, and constrained *ab initio* molecular dynamics (cAIMD), to map energy landscapes for chloride migration in pristine and alloyed LaOCl.

Informed by single dopant correlations, Ca coalloying with Mg and Sr is further used to modulate defect concentrations, and the resulting defect–conductivity correlations are analyzed within a collective-dopant formalism that explicitly includes interaction terms between alloying species. Through this integrated approach, we aim to contrast the relative efficacy of Mg, Ca, and Sr as site-selective dopants for enhancing chloride-ion transport in LaOCl; develop crystal lattice structural descriptors that capture how alloying reshapes lattice compliance and phonon interactions based on Raman spectroscopy; elucidate the connection between local distortion energetics and macroscopic conductivity using cAIMD simulations; and demonstrate how coalloying can be utilized to navigate a multidimensional defect space. The insights that emerge provide design principles for LaOCl-based chloride-ion conductors and point toward more general strategies for engineering anion transport in layered oxyhalides.

## Experimental Section

### Materials

La_2_O_3_ (≥99.9%), NH_4_Cl (≥99.9%), (COO)_2_Ca·H_2_O (≥99.0%), (COO)_2_Mg·2H_2_O (≥99.0%), (COO)_2_Sr·2H_2_O (≥99.0%), and (COO)_2_Ba·2H_2_O (≥99.0%) were purchased from Millipore Sigma. Dy_2_O_3_ (≥99.9%) was purchased from ThermoFisher Scientific. All precursors were dried under a steady flow of N_2_ overnight before use.

### Synthesis

LaOCl powders were prepared using a solid-state reaction by reacting stoichiometric amounts of La_2_O_3_ and NH_4_Cl by adapting a previous method reported in the literature as per^19,20^

1 $$La_{2}O_{3}\left(s\right)+2NH_{4}Cl\left(s\right)\to 2LaOCl\left(s\right)+2NH_{3}\left(g\right)+H_{2}O\left(g\right)$$

Site-selective modification was achieved by supplanting La_2_O_3_ with various molar ratios of the precursors to be substituted in the cation sublattice. For instance, (COO)_2_Ca·H_2_O was used for aliovalent substitution. In an example procedure to prepare powders of Ca_*x*_La_1–*x*_OCl_1–*x*_, 4.5 mmol of La_2_O_3_, 9 mmol of NH_4_Cl, and 1 mmol of calcium oxalate powder were mixed thoroughly with a mortar and pestle and placed in a covered alumina crucible. The mixtures were heated at a controlled heat rate of 5 °C/min to 400 °C for 2 h to evolve NH_3_ and subsequently to 850 °C for 12 h in an MTI GSL-1700 high-pressure tube furnace under an ambient air environment.

A total of four variants were prepared using (COO)_2_M·*x*H_2_O/La_2_O_3_ (M = Mg, Ca, Sr and Ba) molar ratios of 10% that can be expressed in the Kroger—Vink notation as ${\left(M_{La}^{\prime }\right)}_{x}{{\left(\mathrm{La}\right)}_{\mathrm{La}}}_{1-x}O{{\left(\mathrm{Cl}\right)}_{\mathrm{Cl}}}_{1-x}{{\left(V\right)}_{\mathrm{Cl}}^{\cdot }}_{x}$ (M = Mg, Ca, and Sr) as per

2 $$\left(1-x\right)La_{2}O_{3}\left(s\right)+\left(2-2x\right)NH_{4}Cl\left(s\right)+\left(2x\right){\left(COO\right)}_{2}M\left(s\right)+xO_{2}\left(g\right)\to 2La_{1-x}Ca_{x}OCl_{1-x}\left(s\right)+\left(2-2x\right)NH_{3}\left(g\right)+\left(1-x\right)H_{2}O\left(g\right)+4xCO_{2}\left(g\right)$$

The Dy-alloyed samples were prepared in a similar method using a reaction temperature of 1050 °C instead of 850 °C with Dy_2_O_3_ as the precursors. To prepare LaOCl doped with 1 mol % Dy on the La sublattice, 0.05 mmol of Dy_2_O_3_, 4.45 mmol of La_2_O_3_, 9 mmol of NH_4_Cl, and 1 mmol of calcium oxalate were thoroughly mixed using a mortar and pestle. The resulting mixture was transferred to a covered alumina crucible and subjected to heat treatment in an MTI GSL-1700 high-temperature tube furnace under ambient air. The sample was heated at a controlled rate of 5 °C/min to 400 °C and held for 2 h to facilitate NH_3_ release, followed by heating to 1050 °C and holding for 12 h to complete the reaction, yielding compositions that can be expressed in the Kroger-Vink notation as ${\left(M_{La}^{\prime }\right)}_{x}{{\left(N\right)}_{\mathrm{La}}}_{y}{{\left(\mathrm{La}\right)}_{\mathrm{La}}}_{1-x-y}O{{\left(\mathrm{Cl}\right)}_{\mathrm{Cl}}}_{1-x}{{\left(V\right)}_{\mathrm{Cl}}^{\cdot }}_{x}$ (*M* = Mg, Ca, and Sr, N = Dy) as per

3 $$\left(1-x-y\right)La_{2}O_{3}\left(s\right)+yN_{2}O_{3}\left(s\right)+\left(2-2x-2y\right)NH_{4}Cl\left(s\right)+\left(2x\right){\left(COO\right)}_{2}M\left(s\right)+xO_{2}\left(g\right)\to 2La_{1-x-y}N_{y}M_{x}OCl_{1-x}\left(s\right)+\left(2-2x-2y\right)NH_{3}\left(g\right)+\left(1-x-y\right)H_{2}O\left(g\right)+4xCO_{2}\left(g\right)$$

The coalloyed samples were prepared in a similar route using the reaction temperature of 1050 °C with (COO)_2_Ca·H_2_O as the primary precursor and (COO)_2_Mg·2H_2_O and (COO)_2_Sr·2H_2_O as alloying precursors. To prepare coalloyed LaOCl with 10 mol % Ca and 1 mol % Mg on the La lattice, 4.5 mmol of La_2_O_3_, 8.9 mmol of NH_4_Cl, 1 mmol of calcium oxalate and 0.1 mmol of magnesium oxalate were thoroughly mixed using a mortar and pestle. The resulting mixture was transferred to a covered alumina crucible and subjected to heat treatment in an MTI GSL-1700 high-temperature tube furnace under ambient air. The sample was heated at a controlled rate of 5 °C/min to 400 °C and held for 2 h to facilitate NH_3_ release, followed by heating to 1050 °C and holding for 12 h to complete the reaction, yielding compositions that can be expressed in the Kroger-Vink notation as ${\left(M_{La}^{\prime }\right)}_{x}{{\left(N\right)}_{\mathrm{La}}}_{y}{{\left(\mathrm{La}\right)}_{\mathrm{La}}}_{1-x-y}O{{\left(\mathrm{Cl}\right)}_{\mathrm{Cl}}}_{1-x}{{\left(V\right)}_{\mathrm{Cl}}^{\cdot }}_{x}$ (*M* = Ca, *N* = Mg and Sr) as per

4 $$\left(1-x-y\right)La_{2}O_{3}\left(s\right)+\left(2-2x-2y\right)NH_{4}Cl\left(s\right)+\left(2x\right){\left(COO\right)}_{2}M\left(s\right)+\left(2y\right){\left(COO\right)}_{2}N\left(s\right)+\left(x+y\right)O_{2}\left(g\right)\to 2La_{1-x-y}N_{y}M_{x}OCl_{1-x-y}\left(s\right)+\left(2-2x-2y\right)NH_{3}\left(g\right)+\left(1-x-y\right)H_{2}O\left(g\right)+4xCO_{2}\left(g\right)$$

All recovered powders were milky white, fine, and free-flowing. No visible color change was observed for the alloyed samples, irrespective of the alloying element. All products were stored in a nitrogen box.

## Characterization

Powder X-ray diffraction (XRD) patterns were acquired using a Bruker-AXS D8 Vario X-ray powder diffractometer with a Cu Kα radiation source (λ=1.5418Å) in the 2θ range from 5 to 90° at a step size of 0.003°. Rietveld refinements of powder XRD patterns were performed using GSASII.^21^ To quantify alloying-induced local distortion around La centers, a bond-length distortion index was calculated for nominally eight-coordinated La-centered polyhedra using refined interatomic distances inferred from Rietveld refinements. The distortion index was defined as

5 $$D=\left(\frac{1}{n}\right)\overset{n}{\underset{i=1}{\sum }}\frac{\left|l_{i}-\mathrm{l̅}\right|}{\mathrm{l̅}}$$

where *n* = 8, *l*_*i*_ is an individual La–anion bond length in the selected coordination shell, and *l̅* is the average of the eight La–anion bond lengths. For the alloyed compositions, the effective eight-coordinate environment was defined using the eight shortest La–anion contacts, excluding the next layer longest La–Cl contact from the formally nine-coordinated La environment.

Neutron powder diffraction (NPD) pattern was collected at the high-resolution powder diffractometer for thermal neutrons (HRPT) at the Swiss Spallation Neutron Source (SINQ), Paul Scherrer Institute (PSI), Switzerland. Approximately 2.0 g of the sample was packed in a vanadium can with 6 mm outer diameter in a controlled environment under Ar gas atmosphere. NPD was collected using a neutron wavelength of 1.494 Å in the intermediate resolution mode of the instrument.^22,23^ An oscillating radial collimator suppressed Bragg peaks from the sample environment to increase quality of the measurement.^22^ Rietveld corefinement of the NPD and powder XRD patterns were performed with the GSAS II software using its internal tables for neutron scattering lengths and form factors.^21^

Scanning electron microscopy (SEM) was performed using a JEOL JSM-7500F ultrahigh-resolution field-emission instrument with a low-aberration conical objective lens and a cold cathode UHV field-emission conical anode gun. SEM images were acquired at a working distance of 8 mm, accelerating voltage of 20.0 keV, an emission current of 20 μA, and a probe current set at 12 nA/cm^2^. The samples were dispersed on carbon tape and mounted onto standard SEM sample holders. An Oxford EDS system equipped with X-ray and digital imaging was used for elemental mapping.

Elemental concentrations of Dy, La, Ca, and Cl were determined by comparator instrumental neutron activation analysis (INAA). Aliquots of 1–2 mg of powder samples were weighed and transferred into precleaned low density polyethylene (LDPE) irradiation vials. Multielement calibrators were prepared from high-purity La_2_O_3_ (Johnson Matthey, 99.9%) and from dried aliquots of aqueous Dy, La, Mg, Ca, Sr, and Cl solution standards (Inorganic Ventures, ISO 17025 certified). Approximately 0.7 g of high-purity graphite powder was added to each vial, and the irradiation vials were heat-sealed with a soldering iron. The contents of each vial were agitated by rolling and tilting the vials. Neutron irradiations of 30 s were performed using the Texas Engineering and Experiment Station 1 MW TRIGA reactor at a nominal thermal neutron fluence rate of 9.1 × 10^12^ cm^–2^ s^–1^. Following 270 s decay intervals, gamma-ray spectra were acquired for 500 s using an HPGe detector. The data reduction was performed using the NAA software package from Canberra Industries.

XEOL and XANES spectra were acquired at the VLS-PGM beamline at the Canadian Light Source^24^ using the beamline high-energy grating (*E*/Δ*E* > 10 000). Undoped and alloyed powder samples were mounted onto the BL sample holder using double-sided carbon tape and then introduced into the BL experimental chamber. Total fluorescence yield (FLY) XANES and XEOL data were acquired concurrently, all measurements were performed at room temperature and at a pressure better than 1 × 10^–8^ Torr. Spectra were normalized to the intensity of the photon beam measured as the nickel-mesh (90% transmission) drain-current. The mesh is situated just upstream the sample. XANES spectra were acquired in the energy range 90—170 eV with a step size of 0.1 eV and 1 s dwell time, the FLY signal was recorded using a microchannel plate detector.^25^ The partial luminescence data were collected using an Ocean Optics QE65000 spectrometer over the 350–750 nm range. The XEOL spectra, recorded at fixed photon energies, were collected with a dwell time of 5 s, and over a new sample position to minimize radiation damage. Characteristic luminescence signatures of divalent Dy^2+^ species are not detectable in XEOL spectra (*vide infra*).

Temperature-variant Raman spectroscopy measurements were performed on a Horiba XploRA plus Raman microscope with a 532 nm laser excitation source set to a power of 2 mW. A Linkam HFS600E-PB4 stage, coupled with a TMS 94 temperature controller, was used to heat the sample from 25 to 400 °C at a rate of 20 °C min^–1^. The experimental environment was maintained under a nitrogen purge (5 mL min^–1^), whereas the stage was water-cooled using a Pike Technology liquid circulator. Raman spectra were collected at target temperatures at 25 °C and at 50 °C intervals between 50 and 400 °C. Before each measurement, the sample was held at the designated temperature for 10 min to ensure thermal equilibrium. All temperature-variant Raman spectra were processed using a uniform baseline correction and peak-deconvolution protocol to ensure quantitative comparability across unalloyed and alloyed LaOCl. For each spectrum, the baseline was removed using polynomial subtraction over a consistent window, followed by nonlinear least-squares fitting with physically constrained peak positions and widths.

To visualize Raman-active Γ-point phonons in LaOCl, the Phonon Web site^26^ (with phonon eigenvectors from the Materials Project)^27^ was used to generate correlated motion visualizations spanning from low-frequency lattice breathing dominated by heavy La motion to higher-frequency internal modes involving lighter Cl and O atoms.

Matrix density was measured by helium pycnometry using a Quantachrome Ultrapyc/UltraFoam instrument. The instrument was allowed to reach thermal equilibrium prior to operation. Ultrahigh-purity, dry He was supplied via a dual-stage regulator and regulated to ∼ 20 psig at the instrument (relief at 25 psig; regulator settings were kept ≤ 22 psig). Sample cells were preweighed, filled with sample to at least 3/4 of the cell volume, and reweighed to determine sample mass by difference; the cell holder was sealed using an O-ring with a light film of vacuum grease. Prior to analysis, samples were conditioned using the instrument’s built-in purge routine (flow, pulse, or optional vacuum purge as appropriate). Measurements were performed at a target pressure of 17.00 psi with automatic equilibration control and 20-run acquisition until deviation was less than 0.001.

Theoretical crystal density was calculated molecular weight from NAA composition (*M*) and refined cell volumes (*V*) using the following equation: *ρ*_*Theoretical*_ = (*Z·M*)/(*N*_*A*_ *·V*) The difference between *ρ*_*Matrix*_ and *ρ*_*Theoretical*_ can be calculated with *Δρ* = (*ρ*_*Matrix*_ *– ρ*_*Theoretical*_ *)*/*ρ*_*Theoretical*_.

## First-Principles Calculations

Geometry optimizations were carried out using DFT as implemented in the Vienna Ab-initio Simulation Package (VASP) for all alloyed LaOCl configurations.^28,29^ Brillouin zone integration was performed using a 6 × 6 × 6 Monkhorst—Pack mesh.^30^ The projector-augmented wave formalism was used to capture electron—ion interactions. Electron exchange and correlation were addressed using the generalized gradient approximation based on the Perdew–Burke–Ernzerhof functional (PAW-GGA-PBE).^31,32^ Electronic self-consistent loop and ionic relaxation loops were adjusted to be <10^–5^ and 10^–4^ eV, respectively.

The cAIMD method implemented in VASP was used to calculate the free energy change based on thermodynamic integration of the free-energy gradient;^33,34^ a user-defined set of coordinates was used to describe the ion migration, and the SHAKE algorithm constrained the system onto the reaction path.^35^ Chloride-ion migration occurs through infrequent, activated processes of short duration; the cAIMD method enabled us to model these processes between known sites within a time simulation window accessible with molecular dynamics. A 0.002 Å/step increment was applied throughout the calculation for the collective variables to drive the exchange reaction. The free-energy gradient integration used the trapezoid rule; the TS^⧧^ location was identified as the point where the free-energy gradient vanishes.

## Electrochemical Measurements

Electrochemical impedance spectroscopy (EIS) was carried out using Gamry ref-620 potentiostats and a BioLogic HTSH-1100 high-temperature sample holder. The powder samples (about 0.6–0.8 g) were pressed into a pellet of 12 mm in diameter and ca. 1 mm in thickness with 8 tons of pressure using an MSE PRO benchtop automatic laboratory press. Next, the pellets were coated with platinum paste from Sigma-Aldrich (99.9%) and sintered at 400 °C for 8 h under N_2_ flow in Thermo Scientific Thermolyne benchtop muffle furnace. The AC conductivity (σ) of the pellet was measured in the frequency between 1 Hz to 13 MHz at temperatures between 25 and 400 °C. The data obtained were processed with EC-lab software to calculate conductivity. The ionic conductivity, σ, was calculated from the fitted resistance using σ = *L*/(*RA*), where *L* is the pellet thickness, *R* is the resistance obtained from equivalent-circuit fitting, and *A* is the electrode contact area. For circular pellets, *A* = *πd* ^2^/4, where *d* represents the pellet diameter. The uncertainty in conductivity was estimated by standard propagation of error from the pellet thickness, and pellet diameter, according to (*Δσ*/σ)^2^ = (*ΔL*/*L*)^2^ + (2*Δd*/*d*)^2^. Next, DC polarization was conducted on the same pellet using the same setup but with a Pt | solid-electrolyte | Pt configuration with the Gamry ref-620 in chronoamperometry mode. A constant DC bias of 100 mV was applied at both room temperature and 400 °C, and the current was recorded as a function of time until a steady-state response was reached. The decay of the transient current arises from ion accumulation at the blocking interfaces, whereas the steady-state current reflects the electronic contribution.

## Results and Discussion

### Synthesis, Structural Characterization, and Defect Structure

Ca-alloyed LaOCl in the range of 5—30 at. % Ca substitution on La sites adopts a tetragonal matlockite PbFCl-type structure with space group *P*4/*nmm*.^14,36^ In this framework, each La^3+^-ion is coordinated by five Cl^–^ ions—four in the upper layer and one in the adjacent layer—and four O^2–^ ions in the lower layer.^9,36^ The crystal structure of unmodified LaOCl derived from Rietveld refinements is sketched in Figure 1A, highlighting the layered PbFCl-type matlockite framework. Rietveld refinements to powder XRD patterns of unalloyed LaOCl are shown in Figure 1B and a magnified view of the refined crystal structure is sketched in Figure 1C. Site-selective aliovalent alloying with Mg, Ca, and Sr solely substitutes La^3+^ at the Wyckoff *2c* position and introduces Cl vacancies within the anion layers.

**Figure 1:**
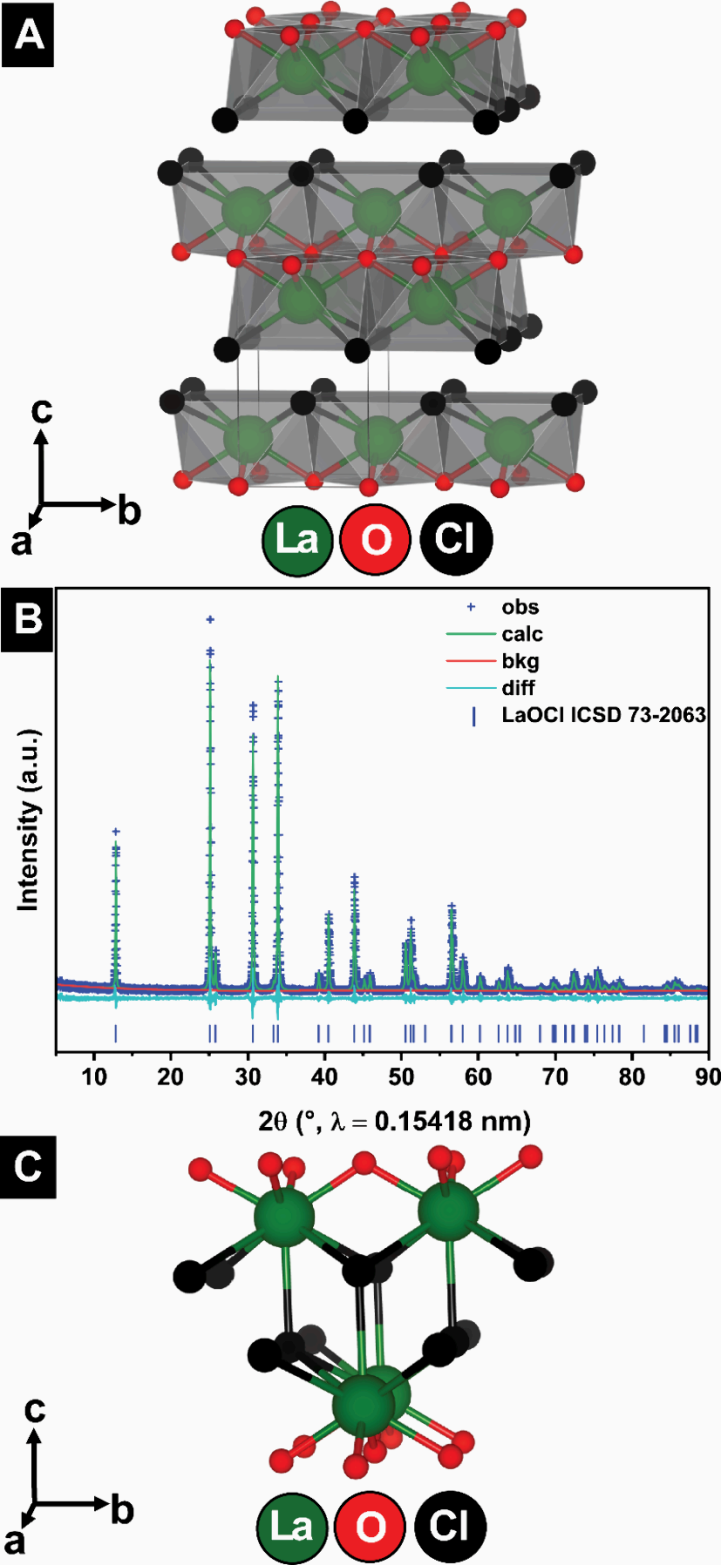
Structural characterization of pristine LaOCl. (A) Crystal structure of LaOCl highlighting layered matlockite framework and anion sublattice. (B) Rietveld refinement to powder XRD patterns for LaOCl. (C) Magnified view of refined crystal structure of LaOCl.

Rietveld refinements to powder XRD of La_0.9_Ca_0.08_OCl_0.92_ (Figure 2A) confirm retention of the matlockite structure despite substantial chloride substoichiometry. Co-refined neutron powder diffraction data (Figure 2B) further supports this conclusion. Given the high neutron scattering length of ^35^Cl, neutron diffraction is especially sensitive to halide occupancies. The X-ray and neutron structures are closely concordant with the latter enabling refinement of the vacancy positions on the chloride sublattice. The corefined crystal structure for the alloyed composition (Figure 2C) shows that the layered framework is preserved upon substitution. The remaining aliovalently alloyed compositions, La_0.9_Mg_0.08_OCl_0.89_, La_0.9_Ca_0.08_OCl_0.92_, and La_0.9_Sr_0.09_OCl_0.93_, are likewise crystallized in the matlockite crystal structure (Figure 2D). In contrast, the attempted Ba-alloyed sample exhibits additional reflections adjacent to 2θ ≈ 23° and 26–32°, which are attributed to BaO and La_2_O_3_, indicating the limited miscibility of Ba^2+^ on the La^3+^ sublattice. The observed limited solubility is consistent with the large mismatch between the Shannon–Prewitt radii^37^ of nine-coordinate La^3+^ (1.216Å) and eight-coordinate Ba^2+^ (1.560Å), which exceeds typical Vegard’s-law tolerances, resulting in a limited range of solid-solution formation. Considering average structure, Figure 2D compares the evolution of *011/101* (2θ = 25°) and 110 (2θ = 30.5°) powder XRD reflections upon alloying; a clear shift to higher 2θ values from unalloyed LaOCl is observed for La_0.90_Mg_0.08_OCl_0.89_, which corresponds to a contraction of the lattice in *c*/*ab* directions, which is consistent with the smaller Shannon radius of Mg in 8-fold coordination (1.03Å). In contrast, the 011/101 (2θ = 25°) and 110 (2θ = 30.5°) powder XRD reflections for La_0.90_Sr_0.09_OCl_0.93_ are shifted to lower 2θ values from unalloyed LaOCl, which reflects lattice expansion along all directions, which is reflective of the higher Shannon radius of 8-fold Sr (1.40Å) as compared to La^3+^ ions.^37^ Notably, however, the Mg-alloyed pattern also exhibits asymmetric and partially overlapping features near 2θ values of 25° and 31°, which are likely derived from gradients in Mg-alloying and microstrain, which bring about reduced X-ray coherent scattering domains rather than a resolvable precursor-derived secondary phase. Such an interpretation is consistent with shifting of the Mg-alloyed reflections to higher 2θ values and is discussed further in the context of alloying-dependent structural distortions (*vide infra*). Since the Shannon radius for 8-fold coordinated Ca (1.26Å) is relatively comparable to that of nine-coordinated La^3+^ (1.216Å), Ca-alloying up to La_0.90_Ca_0.08_OCl_0.92_ results in minimal changes (only a slight contraction) to the lattice parameters and the average structure.^38^

**Figure 2:**
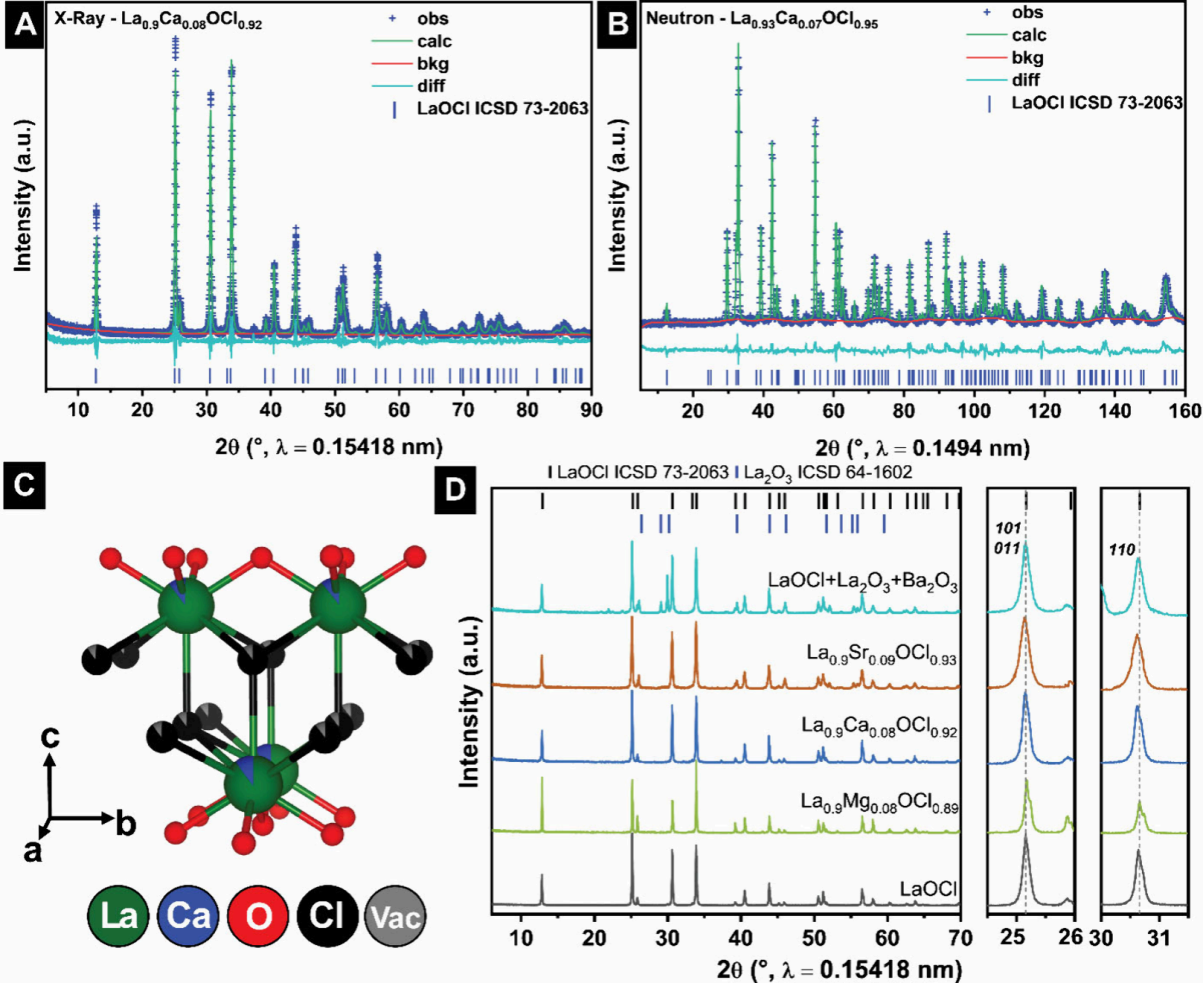
Structural characterization of alloyed LaOCl. (A) Rietveld refinement to powder XRD patterns for La_0.9_Ca_0.08_OCl_0.92_. (B) Neutron diffraction pattern of La_0.93_Ca_0.07_OCl_0.95_. (C) Refined lattice crystal structure for La_0.9_Ca_0.08_OCl_0.92_. (D) Powder X-ray diffraction of La_1–*x*_M_*x*_OCl_1–*x*_ (M = Mg, Ca and Sr) and attempted Ba-alloyed LaOCl.

The chlorine concentration mismatch derives from the preferential exposure of low-energy, Cl-terminated surfaces in LaOCl.^39^ The bulk compositions are obtained from neutron activation analysis (Table S1). Rietveld refinements of powder XRD patterns of La_0.90_Mg_0.08_OCl_0.89_, and La_0.9_0Sr_0.09_OCl_0.93_ are plotted in Figure S1A,B. Refined lattice parameters, atomic positions and thermal parameters are provided in Table S2A–C. Comparison of PXRD and NPD on a common *Q* axis (calculated through *q = 4·π·* sin*θ/λ*; Figure S1C) with indexed *hkl* reflections, affords complementary sensitivity to the heavy-atom framework and light-element sublattice, enabling decoupling of the cation lattice geometry from anion occupancy. Rietveld corefinement of the NPD and powder XRD data corroborates aliovalent alloying via Ca substitution on the La site, yielding refined fractional occupancies of 0.9294 (La) and 0.0706 (Ca) (Table S2D). Consistent with the expected compensation mechanism, refinement of the chloride sublattice yields a fractional occupancy of 0.9536, which evidence the formation of chloride-ion vacancies accompanying Ca incorporation.^40,41^

SEM images and EDS mapping shown in Figures S2—S5 demonstrate the homogeneous alloying of Mg, Ca, and Sr in alloyed LaOCl. No significant changes in particle morphology or dimensions are observed upon alloying with Group II cations as noted in previous work on Ca-alloying of LaOCl.^14^

The selection of Mg^2+^, Ca^2+^, and Sr^2+^ as aliovalent dopants for site-selective modification is guided by their positioning within an acceptable tolerance window of ionic radii in the relevant coordination environments, which enables site-selective substitution on the La^3+^ sublattice while achieving charge compensation through the formation of halide vacancies. This approach preserves the underlying matlockite framework and is further anticipated to promote lattice softening. Importantly, in contrast to many divalent transition metal cations, the alkaline-earth cations are redox-inactive and preserve the primarily ionic bonding motifs, enabling disentanglement of vacancy-mediated transport from electronic and polaronic conduction mechanisms.^8^ Furthermore, p-block divalent cations (e.g., Pb^2+^, Sn^2+^) are avoided owing to the stereochemical expression of 5/6*s* ^2^ electron lone pairs, which can induce pronounced lattice anharmonicity and covalent interactions, further strongly influencing structure–transport correlations.^42^

Electrochemical impedance measurements reveal that site-selective modification with size-matched Group II cations is highly effective. Nyquist plots for densified pellets of alloyed LaOCl powders from 25–400 °C (Figure 3A and Figure S6A–C) exhibit the expected decrease in bulk resistance with increasing temperature. Equivalent-circuit fits using a common circuit (Figure 3B) were used to deconvolute the high- and intermediate-frequency responses from the low-frequency polarization tail as a function of site-selective modification. Because the semicircular features are depressed rather than ideal, these responses were modeled using constant phase elements (CPEs) rather than ideal capacitors (Table S3A–J). Accordingly, the fitted *Q* and *n* parameters are interpreted here as descriptors of nonideal, distributed relaxation processes associated with microstructural heterogeneity, including grain-size distributions, variations in grain boundary structure, surface roughness, local compositional variation, and a distribution of relaxation times, rather than as direct physical capacitances. For this reason, any capacitance-like values inferred from the CPE parameters are treated only as supporting indicators. In the present polycrystalline pellets, both bulk and grain-boundary regions constitute viable pathways for chloride-ion transport; as such, the conductivity values correspond to the combined resistance of the bulk and grain-boundary responses. In other words, even though grain-boundary transport is sensitive to local microstructure and more difficult to modulate independently, the grain-boundary contribution to ion conductivity is embedded in the constant phase elements. By contrast, the low-frequency tail was fitted using a Warburg diffusion element, as its approximately diffusion-like response is consistent with diffusion-controlled/interfacial polarization behavior arising from longer-range ionic redistribution across the pellet/electrode interface and through microstructurally constrained pathways that cannot be adequately represented by purely resistive and capacitive elements alone. Under identical specifications, La_0.9_Ca_0.08_OCl_0.92_ exhibits chloride-ion conductivities that are between three and 4 orders of magnitude higher than pristine LaOCl and roughly 2 orders of magnitude higher than their Mg- or Sr-alloyed counterparts for comparable extents of alloying (Figure 3C).

**Figure 3:**
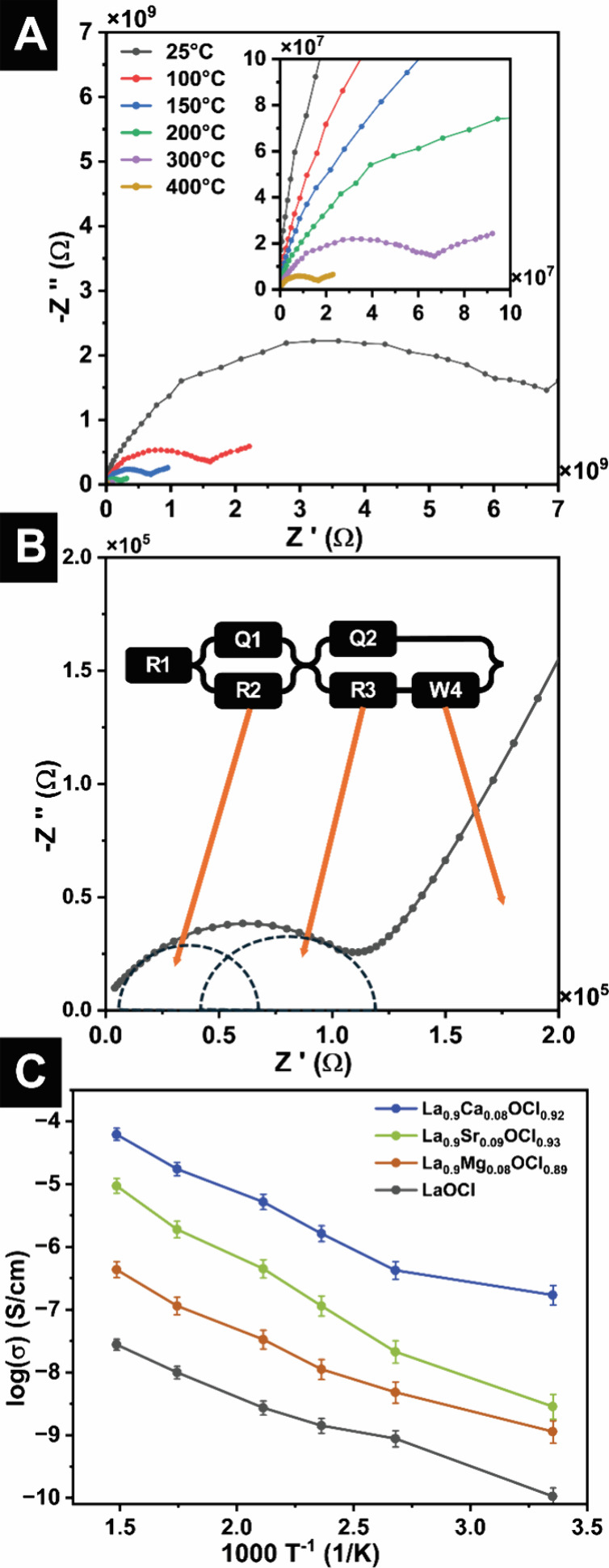
Ion conduction in alloyed LaOCl: (A) Nyquist plot for unalloyed LaOCl from 400 to 25 °C. (B) Equivalent circuits fitting for LaOCl with two constant phase elements (CPE) and one Warburg diffusion element. (C) Temperature-variant ionic conductivity from 400 to 25 °C for LaOCl, La_0.90_Mg_0.08_OCl_0.89_, La_0.90_Ca_0.08_OCl_0.92_, and La_0.90_Sr_0.09_OCl_0.93_.

Notably, the transport trends in Figure 3C are not perfectly Arrhenius-like, indicating that chloride-ion transport in these aliovalently alloyed LaOCl phases is not governed by a single temperature-independent activation process across the full measurement range. In an ideal extrinsic regime with vacancy-dominated transport, aliovalent alloying determines the carrier concentration, and conductivity follows an approximately linear Arrhenius-activated dependence governed primarily by the mobility of the charge carriers. Here, however, the alloyed compositions contain sufficiently high defect concentrations such that diverse combinations of vacancy traps and clusters yield more complex thermal excitation regimes; such traps represent a heterogeneous distributing spanning the range from vacancy–dopant association, vacancy–vacancy interactions, and local defect clustering. At lower temperatures, such short-range interactions can partially immobilize a distribution of the charge carriers and constrain long-range hopping, resulting in a deviation from ideal Arrhenius-like behavior. With increasing temperature, progressive dissociation of these associated defect configurations increases the fraction of effectively mobile carriers and lowers the apparent transport barrier, such that the effective carrier density and the distribution of activation energies is not constant over the full measurement range. At the upper end of the measured temperature regime, the larger defect population can additionally enhance longer-range electrostatic defect–defect interactions (such as through formation of divacancy clusters), further perturbing the ideal dilute-defect Arrhenius limit. Notably, in support of this notion, deviations from Arrhenius-like behavior are more pronounced in the more heavily alloyed materials. As such, increased aliovalent alloying enhances ionic conductivity through increase in extrinsic carrier density and modification of activation energies, but it also increases the probability of defect—defect and defect—lattice interactions.

Viewed through a linearized Arrhenius framework, the measured trends can nevertheless still be parametrized in terms of an effective slope and *y*-intercept, even though the full temperature dependence is not strictly Arrhenius-like. In plots of log­(σ) versus 1000/T, the slope corresponds to the negative effective activation energy, *E*_*a*_, whereas the *y*-intercept corresponds to log­(σ_0_), where σ_0_ represents the effective Arrhenius prefactor (Table S4). Based on this description, the higher conductivity of La_0.9_Ca_0.08_OCl_0.92_ cannot be attributed to the apparent slope or modification of *E*_a_ alone; rather, it reflects a more favorable combination of apparent *E*_*a*_ and, importantly, a markedly larger effective prefactor as compared to the Mg- and Sr-alloyed analogues. As such, the larger *y*-intercept indicates that aliovalent Ca substitution not only facilitates vacancy motion, but also increases the fraction of chloride vacancies that remain effectively mobile and participate in long-range transport. Accordingly, the Arrhenius analysis is consistent with the broader interpretation advanced here that conductivity enhancement is governed by both the energetics of vacancy migration and the extent to which the nominal extrinsic defect population is converted into mobile carriers under finite-temperature conditions as modified by defect/defect or mobile-defect/crystal lattice interactions.

Pellet matrix density, which reflects processing conditions is a critical parameter in evaluating the intrinsic ionic conductivity of ceramic electrolytes.^43^ High matrix density, approaching the theoretical crystallographic density, minimizes open porosity and enhances grain-to-grain contact, thereby reducing tortuous transport pathways and grain-boundary resistance. Conversely, low pellet density introduces interconnected pores that interrupt percolation of the conducting phase.^44,45^ To quantify the true matrix density of our LaOCl-based pellets, we employed helium pycnometry (Table S5). All matrix densities are within 10% of the theoretical crystallographic density, ensuring a valid measurement.

In order to examine defect structure, lattice phonon dynamics, and electronic structure implications of site-selective modification, Figure 4 plots a schematics of the XEOL excitation and relaxation channels alongside La N_4,5_-edge XANES and 3D contour XEOL maps for La_0.90_Dy_0.012_Mg_0.14_OCl_0.92_, La_0.9_Dy_0.013_Ca_0.1_OCl_0.94_, and La_0.9_Dy_0.02_Sr_0.11_OCl_0.93_ (NAA-confirmed compositions are noted in Table S6). In an XEOL measurement, soft X-rays are used to excite La core states, yielding high-energy electron—hole pairs.^18^ The excited states decay through a combination of (i) nonradiative Auger electron ionization (especially pronounced at the giant resonance absorption) and (ii) thermalization of the electron—hole pairs through phonon-mediated processes facilitating energy transfer to adjacent Dy chromophores substituted onto La-sites (Figure 4A).^46,47^ The latter sensitization and relaxation processes activate radiative channels encompassing both intraconfigurational transitions from thermally populated states, as well as transitions from the conduction band edge and defect-related midgap states (Figure 4A).

**Figure 4:**
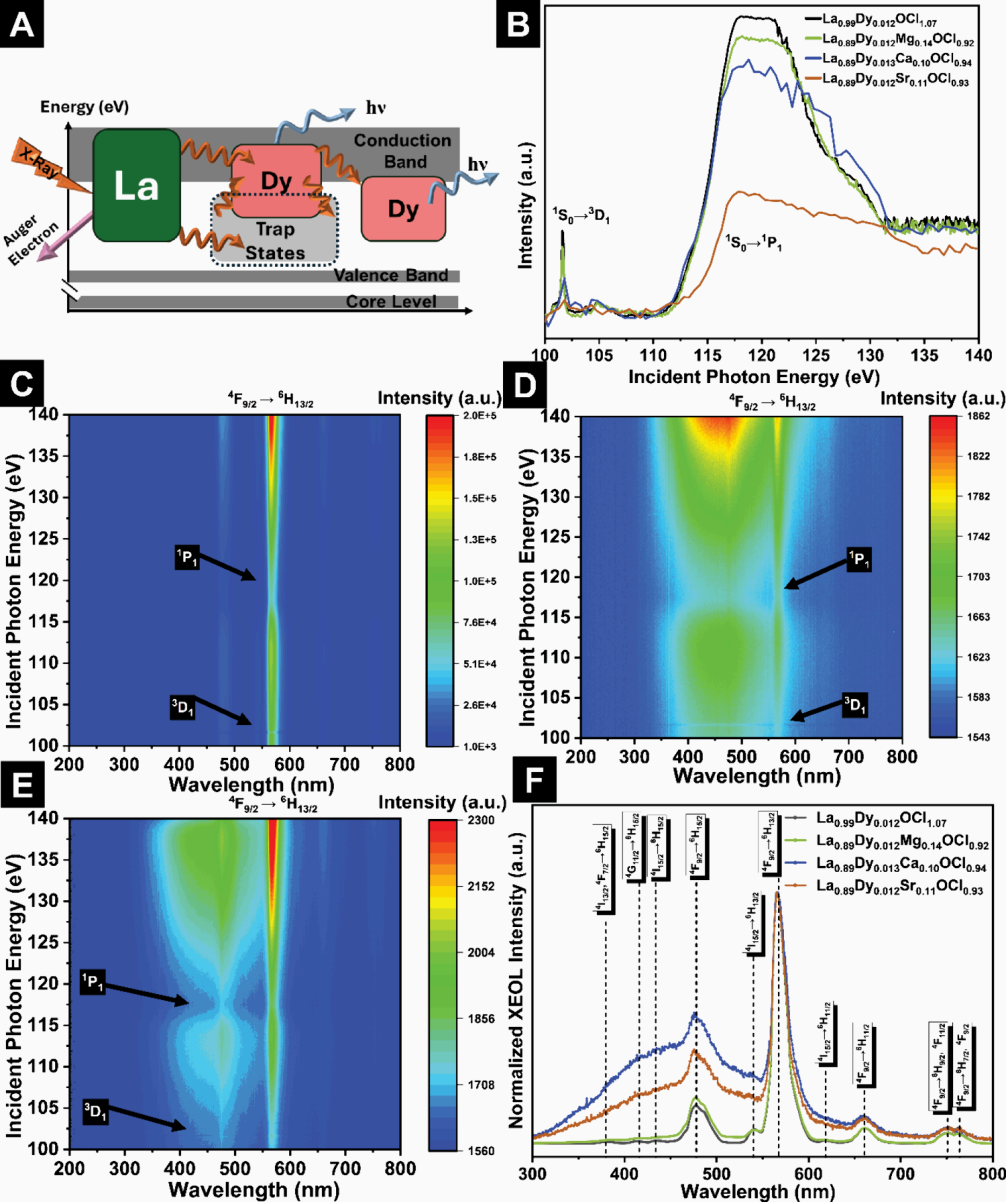
X-ray spectroscopy characterization of the influence of halide vacancies on phonon dispersion and electronic structure. (A) X-ray sensitization mechanism in Dy-alloyed defective LaOCl, showing La absorption, Auger emission, vacancy trapping, energy transfer to Dy, and radiative relaxation. (B) La N_4,5_ edge XANES of La_1‑*x*‑*y*_M_*x*_Dy_*y*_OCl_1–*x*_ (M = Mg, Ca and Sr). 3D contour maps of XEOL signals as a function of incident photon energy for (C) La_0.89_Dy_0.012_Mg_0.14_OCl_0.92_, (D) La_0.89_Dy_0.013_Ca_0.10_OCl_0.94_, and (E) La_0.90_Dy_0.012_Sr_0.11_OCl_0.93_. (F) XEOL spectra of Dy-alloyed defective La_1‑*x*‑*y*_M_*x*_Dy_*y*_OCl_1–*x*_ (M = Mg, Ca and Sr).

Figure 4B contrasts La N_4,5_-edge XANES spectra for La_0.90_Dy_0.012_Mg_0.14_OCl_0.92_, La_0.9_Dy_0.013_Ca_0.1_OCl_0.94_, and La_0.9_Dy_0.02_Sr_0.11_OCl_0.93_ with La_0.99_Dy_0.012_OCl_0.99_. Core-level N_4,5_-edge XANES spectra correspond to La 4*d* → 4*f* excitations. Since La^3+^-ions have a full 4*d* subshell, but empty 5*d* and 4*f* states, a singlet ^1^S_0_ ground state is stabilized. The features marked in the XANES spectra in Figure 3B correspond to 4*d* → 4*f* transitions resulting from the La ^1^S_0_ ground state to (i) a triplet ^3^D_1_ state at ca. 102 eV (which is symmetry forbidden, but observed because of relaxation of selection rules as a result of state-mixing from spin–orbit coupling), and (ii) a singlet ^1^P_1_ state at ca. 120 eV (a symmetry-allowed transition). Considering the mixing of La 4*d* and *4f*-derived states in LaOCl, a giant resonance is observed at ca. 117 eV in Figure 4B with its broad asymmetric shape indicative of a short lifetime.^48^ Figure 4B illustrates that with different alloying elements at the same concentration, the La N_4,5_-edge line shape is markedly altered, reflecting a larger distribution of local La coordination environments (reflective of La sites in proximity of a statistical distribution of halide vacancies),^14,49^ which in turn manifests as a wider energy range where La 4*d*/4*f* mixing is enabled and is reflected in a broader giant resonance. Notably, the distribution of La coordination environments and defect local structure further underpins the distribution of Arrhenius energies and defect interactions observed in EIS data.

Figure 4C—E displays evolution of the XEOL intensity of coalloyed Dy^3+^ chromophores as a function of incident photon energy across the La N_4,5_-X-ray absorption edge. For Dy^3+^-doped LaOCl and for all the aliovalently alloyed compounds, the optical emission intensity is strongly suppressed when the excitation energy coincides with the La giant resonance at ca. 117 eV, in contrast to the higher intensities observed at incident energies just below and above this resonance. The pronounced quenching of luminescence and the underlying radiative relaxation channels signifies the activation of nonradiative Auger-ionization channels at the giant resonance.^10,14,49^ For La_0.99_Dy_0.012_OCl_1.07_ without aliovalent alloying, the suppression of XEOL is confined to a relatively narrow incident energy window (ca. 117–120 eV). Similarly, La_0.89_Dy_0.012_Mg_0.14_OCl_0.92_, which hosts around 10 at. % Mg on the La site, demonstrates a relatively narrow energy range where the XEOL intensity is diminished around the giant resonance. In contrast, the Dy^3+^ XEOL emission bands are suppressed across a much broader range for Ca- and Sr-alloyed LaOCl: 113–120 eV and 115–120 eV, respectively. The strong local electrostatic potentials associated with mobile halide vacancies in these compounds, and their ability to transiently trap ionized Auger electrons, enhance Auger processes at these sites. Auger signatures at the La edge therefore provide a sensitive probe for both local structural distortion and the vacancy-induced reduction in accessible phonon relaxation pathways required for competing sensitization and radiative relaxation channels.

Considering the radiative relaxation channels, away from the giant resonance, core-level excitation of La^3+^ is followed by phonon-mediated thermalization and sensitization of the Dy^3+^ chromophores.^50^ The excited Dy^3+^ chromophores decay through intraconfigurational Dy^3+^ *4f*—*4f* transitions, as well as transitions involving midgap defect states such as those resulting from halide vacancies (Figure 4A). Figure 4F shows XEOL spectra for LaOCl containing 0.012—0.013 at. % Dy as a reporter and 10—14 at. % Mg, Ca, and Sr, acquired under excitation from the La *4d*_3/2_ (N_4_-edge) to *4f* levels. The corresponding Dieke diagram for Dy^3+^ provides a framework for assigning the observed emission bands in Figure 4 and Figure S7A–C. The emergent bands in Figure 4D are unusually broad and cannot be explained solely based on Dy^3+^ *4f—4f* transitions.^51,52^ For the Ca- and Sr-alloyed samples, the full width at half-maximum (fwhm) of bands in the range of 3000–3500 nm approaches ∼ 4500 cm^–1^(0.5 eV), exceeding even the typical 3000—3500 cm^–1^ line widths associated with Ce^3+^ *f—d* transitions. This substantial broadening points to significant contributions from defect-related midgap states in addition to Dy^3+^ intraconfigurational bands as sketched in Figure 4A. Within the Dieke diagram framework (Figure S7C), the blue bands mainly correspond to emissions from thermally populated ^4^I_13/2_,^4^F_7/2,_ and ^4^G_11/2_ manifolds, superimposed on defect-related trap-state emissions, whereas the red channels are dominated by transitions from the thermalized ^4^F_9/2_ state. For unalloyed La_0.99_Dy_0.012_OCl_1.07_, these “hot” bands are strongly suppressed, and the Dy^3+4^F_9/2_ → ^6^H_13/2_ transition at 582 nm is the most intense feature.^14^ However, upon aliovalent alloying, the XEOL response can be viewed as superposition of sharp Dy^3+^ *f—f* lines that dominate the red region of the emission spectrum and broad, defect-mediated bands in blue that are strongly amplified. Although XEOL reveals the emergence of vacancy-derived midgap states upon aliovalent substitution, these states do not appear to add a significant electronic contribution to transport. LaOCl and related rare-earth oxychlorides are wide-bandgap, highly ionic compounds; LaOCl has a reported bandgap of 5.54 eV and an expansive voltage stability window without decomposition or volatilization of halide species.^53^ Prior DFT studies on LaOCl further indicate that vacancy-associated electronic states are strongly localized rather than forming delocalized electronic or polaronic conduction pathways.^54^ This interpretation is further supported by the exceptionally high dielectric breakdown field reported for few-layered LaOCl nanosheets (>10 MV cm^–1^), along with minimal electronic leakage.^55^ Consistent with these reports, DC polarization measurements on both pristine and Ca-alloyed samples using ion-blocking electrodes yield negligible steady-state currents relative to the initial response, supporting predominantly chloride-ion conduction and minimal electronic leakage even upon the introduction of midgap states evidenced by XEOL (Figure S8).

We next turn our attention to probing the evolution of lattice dynamics with site-selective modification. Figure 5A–D presents temperature-variant Raman spectra of unalloyed and aliovalently alloyed LaOCl. To highlight band-center shifts and line shape evolution with increasing temperature, the room-temperature spectrum of each sample is overlaid in a double-line format for direct comparison. The vibrational modes at the Brillouin-zone center for LaOCl have been previously assigned according to the irreducible representation^56,57^

6 $$\Gamma =2A_{1g}+1B_{1g}+3E_{g}+2A_{2u}+E_{u}$$

where the first three terms are Raman-active and the latter two are infrared-active. Consistent with prior reports, the principal features are observed near ca. 125 cm^–1^(La–Cl – A_1g_), 180 cm^–1^(La/Cl–Cl – A_1g_), 205 cm^–1^(Cl–Cl – E_g_), 325 cm^–1^(O–La – E_g_), and 435 cm^–1^(O–La – B_1g_), respectively (see Figure S9A–E for a visualization of lattice phonons contributing to these modes). To interpret the temperature- and alloying-dependent evolution of these bands, it is necessary to connect spectral changes with variations in bond strength, lattice stiffness, and phonon dispersion.^58^ In general, stronger and stiffer bonds exhibit higher vibrational frequencies; accordingly, the ordering of dominant contributions in Figure 5A–D broadly follows O-, Cl-, and La-associated modes in decreasing bond strength.

**Figure 5:**
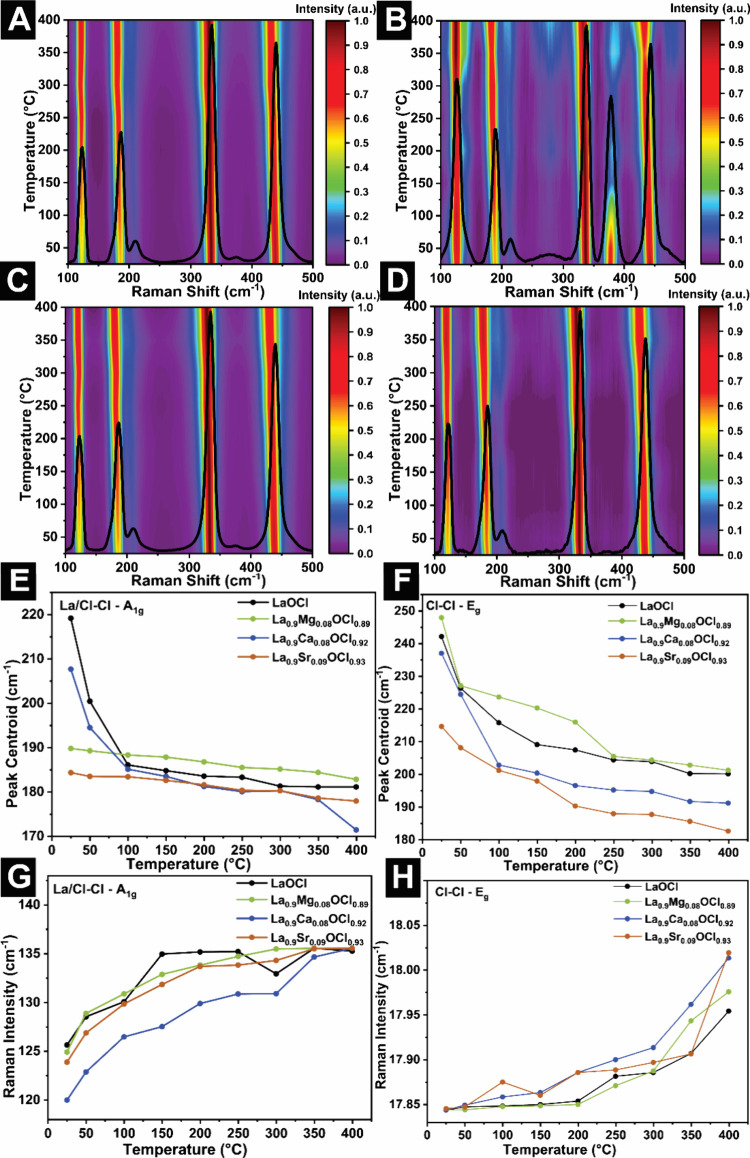
Raman spectroscopy examination of alloying-induced lattice softening and phonon scattering: 3D contour map of Raman modes as a function of temperature for (A) unalloyed LaOCl, (B) La_0.9_Mg_0.08_OCl_0.89_, (C) La_0.9_Ca_0.08_OCl_0.92_, and (D) La_0.9_Sr_0.09_OCl_0.93_. TEC extracted from the temperature evolution of the band centroid (E) La/Cl–Cl – A_1g_ and (F) Cl–Cl – E_g_ modes. Anharmonicity parameter derived from Voigt fits of (G) La/Cl–Cl – A_1g_ and (H) Cl–Cl – E_g_ modes.

In the lowest-energy La–Cl – A_1g_ mode (Figure S9A) the La cations undergo a symmetric breathing-type motion primarily within the *ab*-plane, moving coherently in and out while the O and Cl sublattices remain comparatively rigid; this mode reflects a collective, soft lattice response set by the heavy La mass and relatively shallow potential well. At intermediate frequencies, the Cl-dominated modes are activated: in the La/Cl–Cl – A_1g_ mode (Figure S9B, see also inset to Figure 5E,G), which is examined in some detail, the apical Cl atoms oscillate along the *c*-axis in phase, moving up and down normal to the LaO layers against an essentially immobile La–O framework, whereas in the Cl–Cl – E_g_ mode (Figure S9C, see also inset to Figure 5F,H), the same Cl atoms slide laterally within the *ab*-plane, with neighboring layers moving in opposite directions so that the vibration has a shear-like, doubly degenerate character; these modes probe the stiffness of the La–Cl bonds and the anisotropy of the local potential around the halide sites. The highest energy phonons are O-centered modes owing to the lighter mass of oxygen and the stronger La–O bonding: in the O–La – E_g_ mode (Figure S9D), O atoms move up and down along the *c*-axis with opposite phase between adjacent LaO layers, which yields a layer-breathing-like vibration that modulates the interlayer spacing and electrostatic environment of the halide sublattice. In contrast, in the O–La – B_1g_ mode (Figure S9E), the O atoms slide in opposite directions within the *ab*-plane in neighboring layers, generating an in-plane, antiphase shear distortion.

We specifically examine two quantitative descriptors to capture lattice dynamical changes induced by site-selective modification: a Raman-derived thermal expansion coefficient (TEC) extracted from the temperature-dependent phonon softening, and an anharmonicity parameter derived from systematic line shape analysis of the La/Cl–Cl – A_1g_ and Cl–Cl – E_g_ modes in Figure 5E–H (visualized in Figure S9B,C). These metrics provide a comparative framework for assessing how alloying perturbs lattice stiffness and phonon–phonon/defect scattering in LaOCl. To examine the implications for ion conduction mechanisms, we focus on the Cl-dominated A_1g_ and E_g_ modes, which were fit with Voigt line shapes to account for both instrumental broadening and intrinsic phonon lifetime effects. The fitted peak center (ω) and fwhm (Γ) were extracted at each temperature; uncertainties were obtained from the fit covariance and propagated into all temperature coefficients and derived parameters.

To quantify the effect of alloying on the thermal expansion response of LaOCl, we extracted a Raman-derived thermal expansion parameter from the temperature-dependent softening of the Cl-related A_1g_ and E_g_ phonons. The observed redshift of ω­(*T*) reflects a superposition of quasi-harmonic contributions associated with thermally driven lattice expansion and explicit anharmonic phonon–phonon interactions (Figure 5E–H).^59^ In the absence of composition-specific mode Grüneisen parameters obtained from pressure-dependent Raman or first-principles calibration,^60^ we extract a comparative Raman-based TEC proxy defined from the normalized frequency slope of each mode

7 $$\alpha_{\mathrm{Raman}}^{\left(i\right)}\propto -\frac{1}{\omega_{i}\left(T_{0}\right)}\frac{{d\omega }_{i}}{dT}$$

where *i* denotes A_1g_ or E_g_ and *T*_0_ is a reference temperature, 25 °C in this case. This metric enables direct comparison of alloying-induced changes in lattice softness and thermal response (Tables [Table tbl1A] and [Table tbl1B]).^61^ The A_1g_- and E_g_-derived values were analyzed to assess mode-specific sensitivity and to capture the anisotropic nature of lattice perturbations in layered LaOCl.^62^ Figure 5E,F and Table [Table tbl1A] reveal differences in phonon softening that translate into distinct α_Raman_ trends depending on the nature of divalent alloying. For La/Cl–Cl – A_1g_ (Figure 5E) modes, all compounds show a pronounced initial redshift in the temperature range from 25 to 100 °C, followed by a more gradual evolution at higher temperatures (100—400 °C), which is consistent with a combined quasi-harmonic and anharmonic response.^62,63^ Relative to unalloyed LaOCl, the alloyed compositions exhibit lower centroids of the Raman band, and in some cases, an expanded range of the peak shift across the measured temperature range. Notably, in the Ca-alloyed sample (Figure 5E, blue line), the La/Cl–Cl – A_1g_ mode is shifted by Δν = 13 cm^–1^ (as compared to Δν = 3 cm^–1^ for unalloyed LaOCl, Figure 5E, black line), in the temperature range of 100 — 400 °C, suggesting a larger effective temperature sensitivity of this mode compared to Mg- and Sr-alloyed counterparts.^64^

**1 tbl1A:** Relative Thermal Expansion Coefficients of All Five Raman Modes for Unalloyed LaOCl, La_0.9_Mg_0.08_OCl_0.89_, La_0.9_Ca_0.08_OCl_0.92_, and La_0.9_Sr_0.09_OCl_0.93_

α_Raman_ proxy	Temperature	LaOCl	La_0.9_Mg_0.08_OCl_0.89_	La_0.9_Ca_0.08_OCl_0.92_	La_0.9_Sr_0.09_OCl_0.93_
**La–Cl – A**_**1g**_	25–400 °C	–6.40 × 10^–3^	–1.76 × 10^–3^	–7.53 × 10^–3^	–8.29 × 10^–3^
**La/Cl–Cl – A**_**1g**_	25–100 °C	–0.301	–1.77 × 10^–2^	–0.254	–1.69 × 10^–2^
	100–400 °C	–3.72 × 10^–2^		–3.14 × 10^–2^	
**Cl–Cl – E**_**g**_	25–100 °C	–4.16 × 10^–2^	–3.94 × 10^–2^	–4.72 × 10^–2^	–2.87 × 10^–2^
	100–400 °C	–0.337	–0.364	–0.382	–0.266
**O–La – E**_**g**_	25–400 °C	–1.62 × 10^–3^	–2.71 × 10^–2^	–1.75 × 10^–3^	–2.11 × 10^–2^

**2 tbl1B:** Anharmonicity Parameter of All Five Raman Modes for Unalloyed LaOCl, La_0.9_Mg_0.08_OCl_0.89_, La_0.9_Ca_0.08_OCl_0.92_, and La_0.9_Sr_0.09_OCl_0.93_

*B*_Anharmonicity_ Proxy (°C/cm^–1^)	Temperature	LaOCl	La_0.9_Mg_0.08_OCl_0.89_	La_0.9_Ca_0.08_OCl_0.92_	La_0.9_Sr_0.09_OCl_0.93_
**La–Cl – A**_**1g**_	25–400 °C	5.13 × 10^–6^	–2.11 × 10^–6^	5.80 × 10^–6^	–1.21 × 10^–7^
**La/Cl–Cl – A**_**1g**_	25–400 °C	1.03 × 10^–2^	1.11 × 10^–2^	1.49 × 10^–2^	1.23 × 10^–2^
**Cl–Cl – E**_**g**_	25–200 °C	4.95 × 10^–5^	3.74 × 10^–5^	2.56 × 10^–4^	1.83 × 10^–4^
	200–400 °C	4.79 × 10^–4^	7.42 × 10^–4^	1.02 × 10^–3^	2.30 × 10^–3^
**O–La – E**_**g**_	25–400 °C	–1.30 × 10^–5^	–2.98 × 10^–7^	–1.70 × 10^–5^	1.78 × 10^–6^
**O–La – B**_**1g**_	25–400 °C	4.29 × 10^–6^	1.02 × 10^–5^	3.29 × 10^–5^	–7.04 × 10^–6^

For the Cl–Cl – E_g_ (Figure 5F) mode, the alloy-dependent separation is more apparent as a function of temperature. Raman bands for Sr- and Ca-alloyed samples are lower in frequency as compared to pristine LaOCl and Mg-alloyed LaOCl, which indicates relatively more pronounced alloying-driven softening of this Cl vibration. Taken together, the temperature- and alloying-driven evolution of the two Cl-sublattice-associated modes suggest that Ca and Sr alloying and concomitant introduction of Cl-ion vacancies more strongly reduce the effective lattice stiffness of the La–Cl sublattice (higher |*dω/dT*| and thus larger α_Raman_ proxy) as compared to Mg alloying (Table [Table tbl1A]).^59,60^ Here, reduced lattice stiffness refers to softer effective restoring forces for the Cl-centered vibrations, particularly the in-plane *E*g mode, and thus a more compliant halide framework. Such softening is also consistent with increased phonon entropy and likely contributes to greater ion mobility by better accommodating the local distortions required for chloride-ion hopping.

We define the anharmonicity parameter as the coefficient governing the temperature-driven phonon renormalization and lifetime broadening of the A_1g_ and E_g_ modes extracted from Voigt fitting. Specifically, the temperature dependence of line width was modeled using a standard three-phonon anharmonic decay formalism (Balkanski-type)^65^ as per

8 $$\Gamma \left(T\right)=\Gamma_{0}+B\left[1+\frac{2}{e^{\hbar \omega_{0}/2k_{B}T}-1}\right]$$

where *B* is the anharmonicity parameter for each mode. In alloyed LaOCl, this parameter is best interpreted as an effective anharmonic–disorder descriptor, as line width broadening can include both intrinsic phonon decay and extrinsic scattering from alloy-associated local microstrain and Cl-vacancy disorder.^65^ Using this framework, the extracted *B* values (for both La/Cl–Cl – A_1g_ and Cl–Cl – E_g_ modes, Table [Table tbl1A]) provide a quantitative way to contrast how alloying modifies the phonon scattering landscape in LaOCl. In general, stronger lattice softening in Figure 5E,F is expected to be correlated with enhanced thermal modulation of line widths if alloying increases defect-mediated scattering or strengthens phonon–phonon coupling.^66^ Indeed, Ca- and Sr-alloyed LaOCl show pronounced modulation of line widths and larger B values as compared to unalloyed and Mg-alloyed LaOCl (Table [Table tbl1B]).^59,67^ This coupled view of α_Raman_ and *B* supports the conclusion that alloying modulates not just the effective lattice thermal expansion (which modifies the transition states for migrating Cl-ions) but also modifies phonon interaction/defect scattering strengths along the La–Cl sublattice.

As such, the modulation of lattice dynamics inferred from the Raman-derived TEC proxy and the effective anharmonicity parameter are consistent with the alloying induced trends in ion mobility, with Ca-alloyed LaOCl exhibiting the highest conductivity, followed by Sr-alloyed LaOCl, Mg-alloyed LaOCl, and finally unalloyed LaOCl. The larger temperature sensitivity of the La/Cl–Cl – A_1g_ and Cl–Cl – E_g_ band centers for the Ca- and Sr-alloyed samples suggests a more compliant La–Cl sublattice with softer effective restoring forces and higher vibrational entropy, which can lower the effective migration barrier by facilitating the local distortions needed for vacancy-mediated hopping. The superior performance of the Ca-alloyed composition compared with the Sr analogue likely reflects an optimal balance between ion migration where sufficient lattice softening is achieved without local strain fields or dopant-ion/vacancy association that can trap defects.^68^ In contrast, the comparatively greater lattice stiffness observed for the Mg-alloyed sample, together with its more pronounced disorder-related spectral signatures, is consistent with a scenario in which vacancies are more susceptible to local trapping or short-range ordering, limiting the net transport enhancement obtained from Mg alloying. In summary, these results underscore the importance of alloying and concomitant introduction of vacancies not just for modifying carrier concentration but also for modifying mobility based on modulation of lattice compliance, local lattice distortions accompanying ion migration, and local carrier trapping.^68^

In addition to the Cl-related phonons, the temperature-dependent evolution of the La- and O-associated modes was examined, including La–Cl – A_1g_ (∼125 cm^–1^), O–La – E_g_ (∼325 cm^–1^), and O–La – B_1g_ (∼435 cm^–1^) (Figure S9A,D,E). These modes display the expected thermal softening and relatively modest line width changes upon alloying; however, the alloying-induced variations in band centroids and lineshapes are comparatively more subtle as compared to the La/Cl–Cl – A_1g_ and Cl–Cl – E_g_ modes (Figures S10 and S11, Tables [Table tbl1A] and [Table tbl1B]). As such, the greatest impact of alloying used to assess lattice compliance and phonon interactions are primarily on the Cl sublattice (Figure S9B,C) and these phonons couple strongly to Cl-ion diffusion.

To connect signatures of lattice dynamics to structural origins, we revisit structure solutions determined from Rietveld refinements to specifically examine changes to lattice parameters, atom positions, and the crystal lattice upon aliovalent alloying with Group II cations. The Shannon radii of the aliovalently substituted cations are shown in Figure 6A, where Mg^2+^, Ca^2+^, and Sr^2+^ correspond to 0.89Å, 1.12Å, and 1.26Å, respectively, in 8-fold coordination,^37^ relative to 1.16Å for nine-coordinated La^3+^ ions. Based on Rietveld refinements, the mismatch in ionic radii upon aliovalent substitution substantially modifies La–O–M geometries: the O–La–Cl angle decreases from 122.06° to 114.34° (Mg-alloyed LaOCl), 113.34° (Ca-alloyed), and 115.40° (Sr-alloyed), indicating progressively stronger bending along the alloyed (LaO)^+^ slabs. A complementary out-of-plane distortion metric can be defined; the *z*-axis displacement as sketched in Figure 6A, which reflects the distance along *c*-axis from an unalloyed La site. The *z*-displacement is 0.5153Å in Mg-alloyed LaOCl, 1.3726Å in Ca-alloyed LaOCl, and 1.4810Å in Sr-alloyed LaOCl compared with 1.2622Å for unalloyed LaOCl. To further quantify alloying-induced local distortion around La, we calculated the bond-length-distortion index for nominally eight-coordinated La-centered polyhedra based on the refined interatomic distances. The resulting distortion index follows the trend Ca (0.1046) > Sr (0.1036) > Mg (0.1020), which suggests that site-selective modification with Ca-ions induces the largest deviation of the local La coordination environment. Taken together, these structural metrics show that Ca and Sr alloying more strongly perturb the La–Cl framework as compared to Mg alloying, thereby yielding a more deformable crystal lattice that can better accommodate vacancy-mediated hopping. Ca-alloyed LaOCl exhibits substantial bond-angle distortion, which together with the largest La-centered distortion index is consistent with strong local structural perturbation for chloride-ion transport. The Mg-alloyed sample, despite introducing aliovalent defects, displays the smallest *c*-axis distortion, the most subtle bond-angle deviation from the unalloyed compound, and the lowest La-centered distortion index, which is consistent with preservation of a stiffer and less compliant lattice that more constrains restricts ion mobility at comparable alloying levels. These alloying-induced structural distortions, summarized in Table [Table tbl1A], further rationalize the observed trends in lattice dynamics and their correlation with ion mobility.

**Figure 6:**
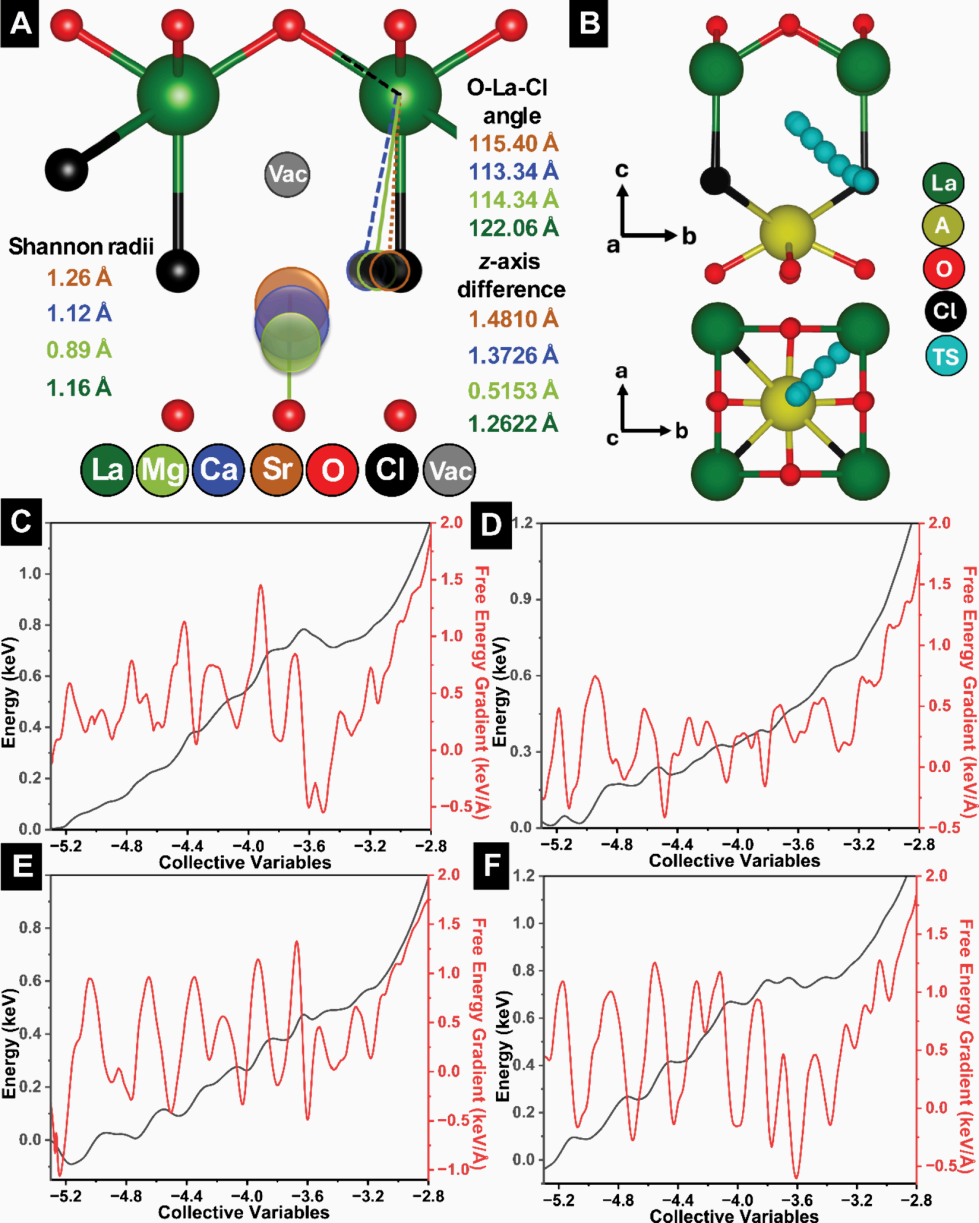
Structure perturbations induced by aliovalent alloying. (A) Schematic of the La–O–M environment (M = Mg, Ca, Sr) showing alloy-dependent Shannon radii, O–La–M bond angles, and *z*-axis displacement differences used to quantify local off-centering and distortion. Lowest energy Cl-ion migration pathway with alloyed LaOCl (TS = transition states, A = alloying element) of around 8 at. % vacancy corresponding to unit cell dimensions of 108 atoms viewed along the (B) crystallographic *a*-axis and *c*-axis. cAIMD free-energy profile and free energy gradient as a function of collective variables for (C) unalloyed LaOCl, (D) La_0.92_Mg_0.08_OCl_0.92_, (E) La_0.92_Ca_0.08_OCl_0.92_, and (F) La_0.92_Sr_0.08_OCl_0.92_.

While structure solutions derived from Rietveld refinements to powder XRD patterns capture the long-range, time-averaged structure, alloying in LaOCl is also expected to introduce local strain, off-centering, and vacancy-coupled distortions that may not be fully resolved in average structural parameters. To bridge this gap between average structure and local dynamical behavior, we examine cAIMD energy landscapes associated with alloy-centered distortion coordinates to examine ion migration pathways^14^ (Figure 6B–F). The effective cAIMD distortion energies decrease from 0.829 eV for unalloyed LaOCl (Video S1) to 0.710 eV for the Mg-alloyed LaOCl (Video S2) and 0.778 eV for the Sr-alloyed environment (Video S3), reaching a minimum of 0.495 eV for the Ca-alloyed environment as shown in Video S4. This ordering indicates that Ca alloying yields the most readily deformable local lattice environment under finite-temperature sampling, consistent with a higher propensity for bond-angle modulation and cation off-centering within the La–Cl framework resolved from Rietveld refinements. Such a cAIMD picture also helps rationalize why the conductivity trends in Figure 3C are not perfectly Arrhenius-like. In an ideal linear Arrhenius regime, transport would be governed by a single temperature-independent migration barrier; however, the alloying-dependent cAIMD distortion energies and free-energy gradients indicate that chloride-ion motion occurs on a migration landscape that is strongly coupled to local lattice deformation and alloy-specific defect environments. Accordingly, the experimental Arrhenius slopes are best interpreted as effective, temperature-averaged activation energies rather than as singular fixed microscopic barriers. Within this framework, the especially low cAIMD distortion energy of the Ca-alloyed environment is consistent with both the much higher conductivity of La_0.9_Ca_0.08_OCl_0.92_ and its more pronounced deviation from ideal linearity, since the same lattice softness that promotes chloride-ion motion also increases access to multiple thermally activated local configurations during transport. The resulting cAIMD energy and free-energy gradient profiles alongside static average structure distortions inferred from Rietveld refinements (Figures 1A and 2A) provide rationalization for the alloying-induced softening of the La/Cl–Cl – A_1g_ and Cl–Cl – E_g_ modes observed in Raman spectroscopy (Figure 5E–H). The distortions to average and local structure underpin pronounced reductions in the energetics of distortions mediating ion conduction. Together, the average structure solutions and lattice dynamics in concert with cAIMD simulations provide a multiscale framework for understanding local distortion energetics and ion conduction.^35,69^

To further enhance chloride-ion transport beyond single-ion Ca substitution, coalloying with a second divalent cation was explored, a strategy commonly employed in solid-state electrolyte design to increase carrier concentration and local structural disorder without introducing additional redox activity.^12,13^ Candidate coalloying elements were restricted to redox-innocent divalent cations, with Mg^2+^ and Sr^2+^ selected because they bracket Ca^2+^ in Shannon radius and thus provide a controlled way to modulate local distortions in the LaOCl framework. Figure 7A shows that introducing ca. 1, 3, and 5 at. % of Mg or Sr in addition to ca. 8 at. % Ca yields a series of coalloyed La_0.9_Ca_0.08_Mg_*x*_OCl_0.83–0.85_ and La_0.9_Ca_0.09_Sr_*y*_OCl_0.85–0.86_ compositions (Table S7) while preserving the LaOCl-type structure type. The magnified views of powder XRD patterns at 2θ = 25–26° and 30–31° exhibit subtle, systematic peak shifts relative to La_0.9_Ca_0.08_OCl_0.92_, consistent with modest adjustments of lattice spacing induced by Mg and Sr coalloying rather than the formation of secondary phases. Rietveld refined crystal structures for all coalloyed samples are shown in Figure S12A–F and detailed refinement parameters are provided in Table S8A–F.

**Figure 7:**
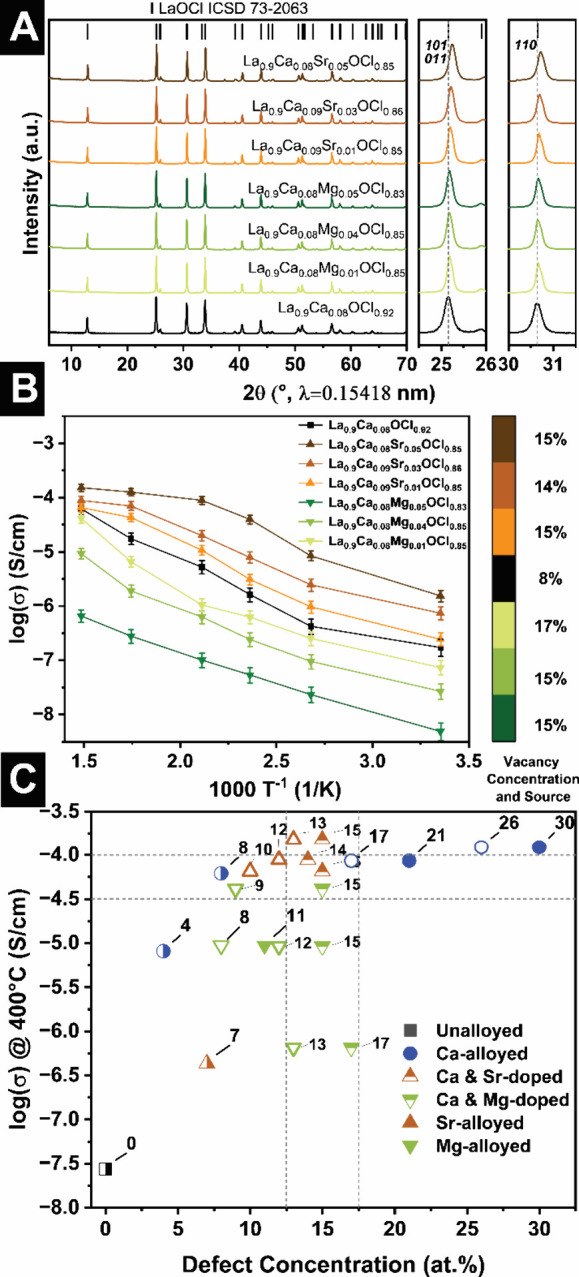
Coalloying of LaOCl to enhance ion conductivity: (A) Powder XRD patterns for La_0.9_Ca_0.08_OCl_0.92_ and Ca-based coalloyed compositions, including La_0.9_Ca_0.08_Mg_0.01_OCl_0.85_, La_0.9_Ca_0.08_Mg_0.04_OCl_0.85_, La_0.9_Ca_0.08_Mg_0.05_OCl_0.83_, La_0.9_Ca_0.09_Sr_0.01_OCl_0.85_, La_0.9_Ca_0.09_Sr_0.03_OCl_0.86_, and La_0.9_Ca_0.08_Sr_0.05_OCl_0.85_. (B) Arrhenius plots of log­(σ) versus 1000/T with color-coded vacancy concentrations, highlighting conductivity enhancement and tuning based on coalloying. (C) log­(σ) at 400 °C versus defect concentration for unalloyed, single-alloyed, and coalloyed samples, showing a nonlinear defect–transport relationship and an optimal defect regime in the design space.

The corresponding Arrhenius plots in Figure 7B demonstrate that Ca-based coalloying yields compounds with conductivities that are comparable to or higher than the Ca-only reference at a given temperature. The color bar indicates that these enhancements correlate with increased halide vacancy concentrations. The Arrhenius plots in Figure 7B exhibit modest deviations from ideal linearity, consistent with the interacting-defect behavior described above for the single-alloyed compositions. In the coalloyed LaOCl phases, this deviation is further amplified because the influence of vacancy–dopant, vacancy–vacancy interactions, and local defect clustering is exacerbated at high concentrations. In addition, collective effects arising from the simultaneous presence of Ca and a second divalent alloying species further perturb the chloride-ion migration landscape and contribute to increased phonon entropy. As a result, transport in the coalloyed compositions is not governed by a single temperature-independent activation energy across the full measurement range. The stronger curvature observed in the more highly alloyed compositions is consistent with this picture: increased coalloying shifts the conductivity upward because of higher extrinsic carrier density, but also increases the probability of collective defect interactions that give rise to non-Arrhenius vacancy transport. Nyquist plots for the coalloyed compounds are shown in Figure S13A–F. A plot of the conductivity measured at 400 °C against the total defect concentration (Figure 7C) yields a nonlinear defect–transport relationship: the conductivity increases sharply from unalloyed LaOCl to Ca- and Ca–Sr/Ca–Mg-containing compositions; however, the conductivity does not continue to rise monotonically with increasing vacancy concentration likely because of increased local trapping and formation of vacancy clusters. Instead, the most conductive samples cluster within an intermediate defect window, suggesting that coalloying improves performance through a combination of (i) increasing the effective charge-carrier concentration by increasing the concentration of halide vacancies and (ii) through enhancements of ion mobility through modulation of lattice compliance. Vacancy concentrations beyond ca. 20 at. % imbue strong local distortions, vacancy clusters, or local traps, particularly in Mg-rich compositions, which constrain further gains in ionic conductivity.

A more formal way to view the Ca–Mg and Ca–Sr coalloying results in Figure 7C is to treat the measured conductivity as a bivariate function of two alloy concentrations or collective alloying/dopant effects, following the collective-dopant framework proposed for codoped VO_2_.^70^ In this picture, the relevant property *M* (here taken as log σ at a fixed temperature) depends on the concentrations of Ca and a second divalent alloying element *M*′ = Mg or Sr as per

9 $$M\left(\left[\mathrm{Ca}\right],\left[M^{\prime }\right]\right)=M_{0}+\Delta M_{\mathrm{Ca}}\left(\frac{\left[\mathrm{Ca}\right]}{{\left[\mathrm{Ca}\right]}_{0}}\right)+\Delta M_{M\prime }\left(\frac{\left[M^{\prime }\right]}{{\left[M^{\prime }\right]}_{0}}\right)+\Delta M_{\mathrm{int}}\left(\left[\mathrm{Ca}\right],\left[M^{\prime }\right]\right)$$

where [Ca]_0_ and [*M*′]_0_ are reference single-alloy concentrations, Δ*M*_Ca_ and Δ*M*_*M′*_ represent the individual (generally nonlinear) contributions of Ca and *M*′ and Δ*M*_int_ captures the additional change arising from their simultaneous presence. A dimensionless interaction factor can then be defined as

10 $$\eta \left(\left[\mathrm{Ca}\right],\left[M^{\prime }\right]\right)=\frac{\Delta M_{\mathrm{int}}\left(\left[\mathrm{Ca}\right],\left[M^{\prime }\right]\right)}{\Delta M_{\mathrm{Ca}}\left(\frac{\left[\mathrm{Ca}\right]}{{\left[\mathrm{Ca}\right]}_{0}}\right)+\Delta M_{M\prime }\left(\frac{\left[M^{\prime }\right]}{{\left[M^{\prime }\right]}_{0}}\right)}$$

with η > 1 indicating cooperative coalloying (the combined effect exceeds the sum of the single-alloy contributions) and η < 1 indicating antagonistic behavior. The conductivity maximum observed for Ca–Sr compositions at intermediate defect concentrations is consistent with a regime where η is close to or slightly above unity, whereas the downturn in Ca–Mg compositions at higher defect levels suggests η < 1, i.e., additional Mg begins to introduce trapping or percolation bottlenecks that offset the benefits of increased vacancy concentration. This framework emphasizes that the optimum in Figure 6C arises from a balance between the individual alloying effects of Ca and the coalloying element, and their collective interaction encoded in Δ*M*_int_ and η rather than from carrier concentration alone

## Conclusions

In this Article, we demonstrate that site-selective aliovalent alloying of LaOCl with divalent alkaline-earth ions is an effective strategy to enhance chloride-ion conductivity. Substitution of La^3+^ with Mg^2+^, Ca^2+^, and Sr^2+^ yields La_1–*x*_M_*x*_OCl_1–*x*_ solid solutions with charge-compensating chloride vacancies while preserving the quasi-layered PbFCl-type matlockite framework. Among the dopants examined, Ca is the most effective, yielding the largest conductivity enhancement relative to unalloyed LaOCl, whereas Sr yields more modest improvements and Mg is least effective. The origin of these differences arises not from vacancy concentrations alone, but are underpinned by the magnitude of local structural distortion, lattice dynamics (specifically softening of phonons), and energetics of trapping of mobile carriers by defects and dopants.

X-ray and neutron powder diffraction, together with compositional analysis, confirm successful aliovalent substitution and chloride-vacancy formation. Variable-temperature Raman spectroscopy shows that Ca- and Sr-alloying bring about more extensive softening of Cl-centered phonon modes and greater anharmonicity as compared to Mg-alloying, which is consistent with a more compliant La–Cl sublattice that better accommodates vacancy-mediated hopping. Rietveld-derived structural descriptors further show that Ca- and Sr-containing compositions exhibit larger deviations in O–La–Cl bonding geometry and stronger out-of-plane cation displacements, indicating that these alloying ions more effectively reshape the local environment surrounding mobile chloride ions.

First-principles calculations corroborate that site-selective modification modifies both vacancy energetics and migration landscapes. In particular, Ca-containing compositions most effectively flatten the free-energy landscape for chloride-ion migration, consistent with the experimentally observed conductivity maximum. Co-alloying studies further reveal that ionic conductivity does not increase monotonically with defect concentration. Instead, the most conductive compositions lie within an intermediate defect regime, indicating that increasing vacancy concentration is beneficial only until defect association, local trapping, or vacancy clustering begin to offset gains in mobility. Taken together, these results establish that high chloride-ion conductivity in LaOCl emerges from a balance between modulation of lattice dynamics, modification of the energetics of Cl-ion migration pathways, and introduction of chloride vacancies. More broadly, this work provides a design framework for halide-ion conductors in which site-selective modification is used not only to introduce vacancies, but also to tune lattice compliance, local distortions, and defect interactions to facilitate fast anion transport.

## Supplementary Material











## Notes

cAIMD video of chloride-ion migration in unalloyed and alloyed LaOCl. The Supporting Information is available free of charge at https://pubs.acs.org/doi/10.1021/acsaem.6c00392.

Supplementary powder X-ray and neutron diffraction refinements; SEM and EDS maps; XEOL and Raman supplementary spectra; Nyquist plots, density data, and compositional and refinement tables; coalloyed structural refinements; and phonon visualizations (PDF)
Cl-ion migration pathway derived from cAIMD simulations of unalloyed LaOCl (MP4)
Cl-ion migration pathway derived from cAIMD simulations of La_0.92_Mg_0.08_OCl_0.92_ (MP4)
Cl-ion migration pathway derived from cAIMD of La_0.92_Sr_0.08_OCl_0.92_ (MP4)
Cl-ion migration pathway derived from cAIMD of La_0.92_Ca_0.08_OCl_0.92_ (MP4)
