# Supplementary material for: Site-Selective Modification of Lanthanum Oxychloride to Modulate Halide-Ion Conduction
Source: ACS Appl Energy Mater. 2026 Apr 16;9(8):5265–82. doi: 10.1021/acsaem.6c00392 (PMC13126442; doi:10.1021/acsaem.6c00392)
Supplement: Supplementary file 1 [file ae6c00392_si_001.pdf]

Supporting Information

Site-Selective Modification of Lanthanum Oxychloride to Modulate Halide-Ion Conduction

*Jingxiang Cheng,[1,2] Victor Alexander Gomez,[3,4] Alice R. Giem,[1,2] Carlos A. Larriuz,[1,2] Samuel Franz Gatti,[5,6] Adrian F. Silva,[7] Lucia Zuin,[8] Sigita Trabesinger,[5] and Sarbajit Banerjee[2,3,4]**

[1]Department of Chemistry, Texas A&M University, College Station, TX 77843-3012, United States
[2]Department of Material Science and Engineering, Texas A&M University, College Station, TX 77843-3012, United States
[3]Laboratory for Inorganic Chemistry, Department of Chemistry and Applied Biosciences, ETH Zurich, Vladimir-Prelog-Weg 2, CH-8093 Zürich, Switzerland
[4]Laboratory for Battery Science, PSI Center for Energy and Environmental Sciences, Paul Scherrer Institute, Forschungsstrasse 111, CH-5232 Villigen PSI, Switzerland
[5]PSI Center for Energy and Environmental Sciences, Paul Scherrer Institute, Forschungsstrasse 111, CH-5232 Villigen PSI, Switzerland
[6]School of Engineering, École Polytechnique Fédérale de Lausanne (EPFL), Lausanne 1015, Switzerland
[7]Department of Nuclear Engineering, Texas A&M University, College Station, TX 77843-3012, United States
[8]Canadian Light Source, University of Saskatchewan, Saskatoon, SK S7N 2V3, Canada
Correspondence: sbanerje@ethz.ch

Table S1: Molar ratio of all precursors used, expected composition considering a stoichiometric reaction, and actual product composition of Mg, Ca and Sr-alloyed products as determined by NAA.

**Precursors (Mole Ratio) (M = Mg, Ca & Sr)**			**Expected product composition**	**Actual product composition**
$(1-x)La_2O_3$	$2x(COO)_2Mg$	$(2-2x)NH_4Cl$	$La_{1-x}Mg_xOCl_{1-x}$	$La_{1-x}Mg_xOCl_{1-x}$
0.9	0.2	1.8	$La_{0.9}Mg_{0.1}OCl_{0.9}$	$La_{0.9}Mg_{0.08}OCl_{0.89}$
$(1-x)La_2O_3$	$2x(COO)_2Ca$	$(2-2x)NH_4Cl$	$La_{1-x}Ca_xOCl_{1-x}$	$La_{1-x}Ca_xOCl_{1-x}$
0.9	0.2	1.8	$La_{0.9}Ca_{0.1}OCl_{0.9}$	$La_{0.9}Ca_{0.08}OCl_{0.92}$
$(1-x)La_2O_3$	$2x(COO)_2Mg$	$(2-2x)NH_4Cl$	$La_{1-x}M_xOCl_{1-x}$	$La_{1-x}M_xOCl_{1-x}$
0.9	0.2	1.8	$La_{0.9}Sr_{0.1}OCl_{0.9}$	$La_{0.9}Sr_{0.08}OCl_{0.9}$

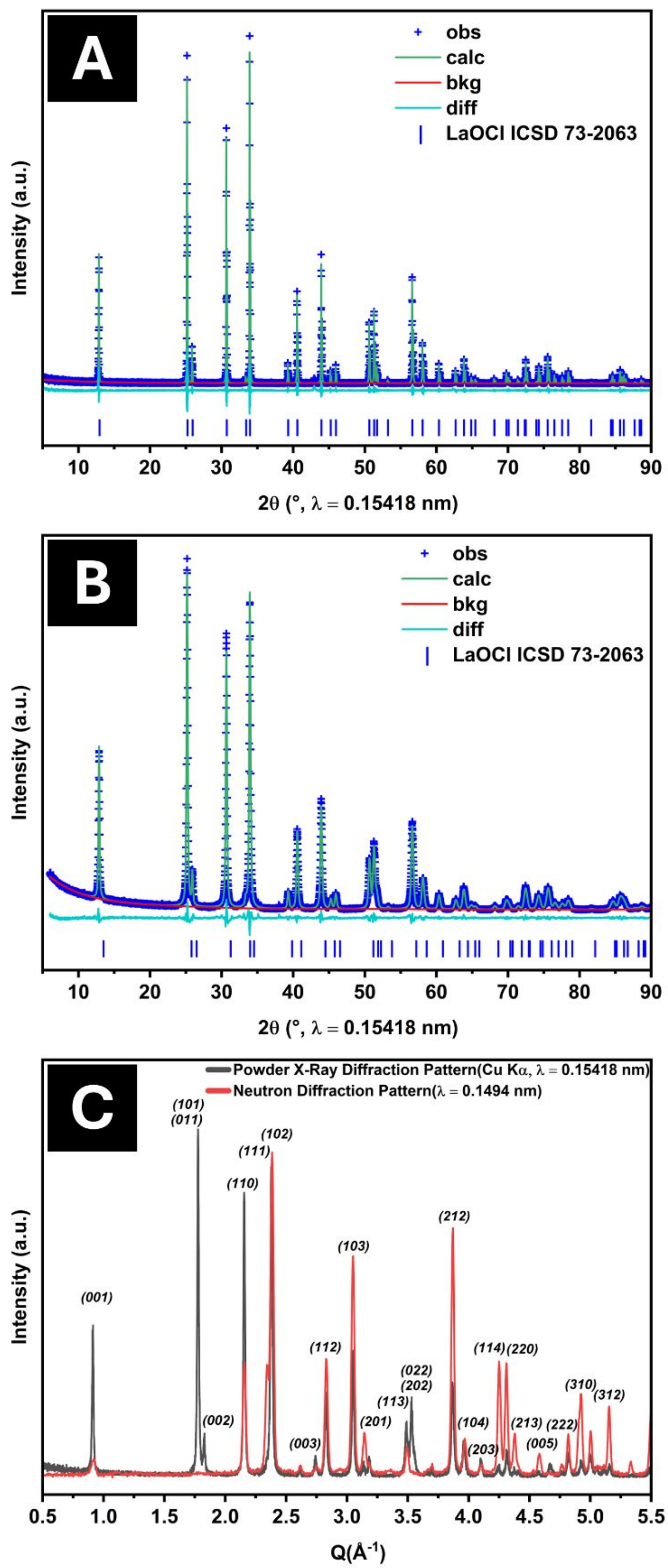

Figure S1: Rietveld refinements of powder XRD patterns indexed to ICSD 73-2063 of (A) $La_{0.90}Mg_{0.08}OCl_{0.89}$; and (B) $La_{0.90}Sr_{0.09}OCl_{0.93}$; (C) Comparison of PXRD and NPD indexed to ICSD 73-2063.

Table S2A: Refined PXRD lattice parameters, atomic positions and thermal parameters for $La_{0.9}Mg_{0.08}OCl_{0.89}$.

$La_{0.9}Mg_{0.08}OCl_{0.89}$						
wR	10.44%	R	7.60%	χ^2	2.96	
2θ range	6 - 90°	Radiation	Cu Kα 1.5406 Å	Temp	295 K	
Formula	$La_{1-x}Mg_xOCl_{1-x}$	Z	2	V (Å³)	116.287	
a=b (Å)	4.1147(8)	*c (Å)*	6.8684(9)	S.G.	*P4/nmm*	
Atom	*x*	*y*	*z*	frac	Wyckoff	Uiso
La	0.0019(4)	0.5034(2)	0.1778(9)	0.90	2c	0.0043(6)
Mg	0.9996(7)	0.5051(3)	0.1738(1)	0.08	2c	0.0037(5)
O	0.9994(7)	0.0001(6)	0.0028(9)	1	2a	0.0124(5)
Cl	0.0011(6)	0.4988(5)	0.6315(8)	0.89	2c	0.0109(6)

Table S2B: Refined PXRD lattice parameters, atomic positions and thermal parameters for $La_{0.9}Ca_{0.08}OCl_{0.92}$.

$La_{0.9}Ca_{0.08}OCl_{0.92}$						
wR	10.85%	R	7.91%	χ^2	2.70	
2θ range	6 - 90°	Radiation	Cu Kα 1.5406 Å	Temp	295 K	
Formula	$La_{1-x}Ca_xOCl_{1-x}$	Z	2	V (Å³)	116.641	
a=b (Å)	4.1182(5)	*c (Å)*	6.8776(6)	S.G.	*P4/nmm*	
Atom	*x*	*y*	*z*	frac	Wyckoff	Uiso
La	0.0018(7)	0.5009(4)	0.1725(8)	0.90	2c	0.0053(3)
Sr	0.0039(3)	0.4965(1)	0.1723(8)	0.08	2c	0.0061(6)
O	0.0002(3)	0.0006(5)	0.0021(2)	1	2a	0.0149(6)
Cl	0.0009(3)	0.4997(6)	0.6331(7)	0.92	2c	0.0125(5)

Table S2C: Refined PXRD lattice parameters, atomic positions and thermal parameters for $La_{0.9}Sr_{0.08}OCl_{0.93}$.

$La_{0.9}Sr_{0.08}OCl_{0.93}$						
wR	10.64%	R	7.76%	χ^2	2.44	
2θ range	6 - 90°	Radiation	Cu Kα 1.5406 Å	Temp	295 K	
Formula	$La_{1-x}Sr_xOCl_{1-x}$	Z	2	V (Å³)	117.005	
a=b (Å)	4.1218(5)	*c (Å)*	6.8870(6)	S.G.	*P4/nmm*	
Atom	*x*	*y*	*z*	frac	Wyckoff	Uiso
La	0.0018(1)	0.5018(7)	0.1760(2)	0.90	2c	0.0036(2)
Sr	0.0030(5)	0.4977(2)	0.1719(7)	0.08	2c	0.0075(7)
O	0.9980(6)	0.9999(3)	0.0017(1)	1	2a	0.0116(2)
Cl	0.0013(4)	0.5002(3)	0.6327(7)	0.93	2c	0.0135(2)

Table S2D: Refined NPD lattice parameters, atomic positions and thermal parameters for $La_{0.92}Ca_{0.07}OCl_{0.95}$.

$La_{0.92}Ca_{0.07}OCl_{0.95}$						
wR	7.169%	R	9.81%	Temp	295 K	
Formula	$La_{1-x}Ca_xOCl_{1-x}$	2θ range	5 - 160°	V (Å³)	116.786	
a=b (Å)	4.1253	*c (Å)*	6.8625	S.G.	*P4/nmm*	
Atom	*x*	*y*	*z*	frac	Wyckoff	
La	0.2500	0.2500	0.1747	0.93	2c	Uiso
Ca	0.2500	0.2500	0.1747	0.07	2c	0.0126
O	0.7500	0.2500	0.0000	1	2a	0.0126
Cl	0.2500	0.2500	0.6276	0.95	2c	0.0175

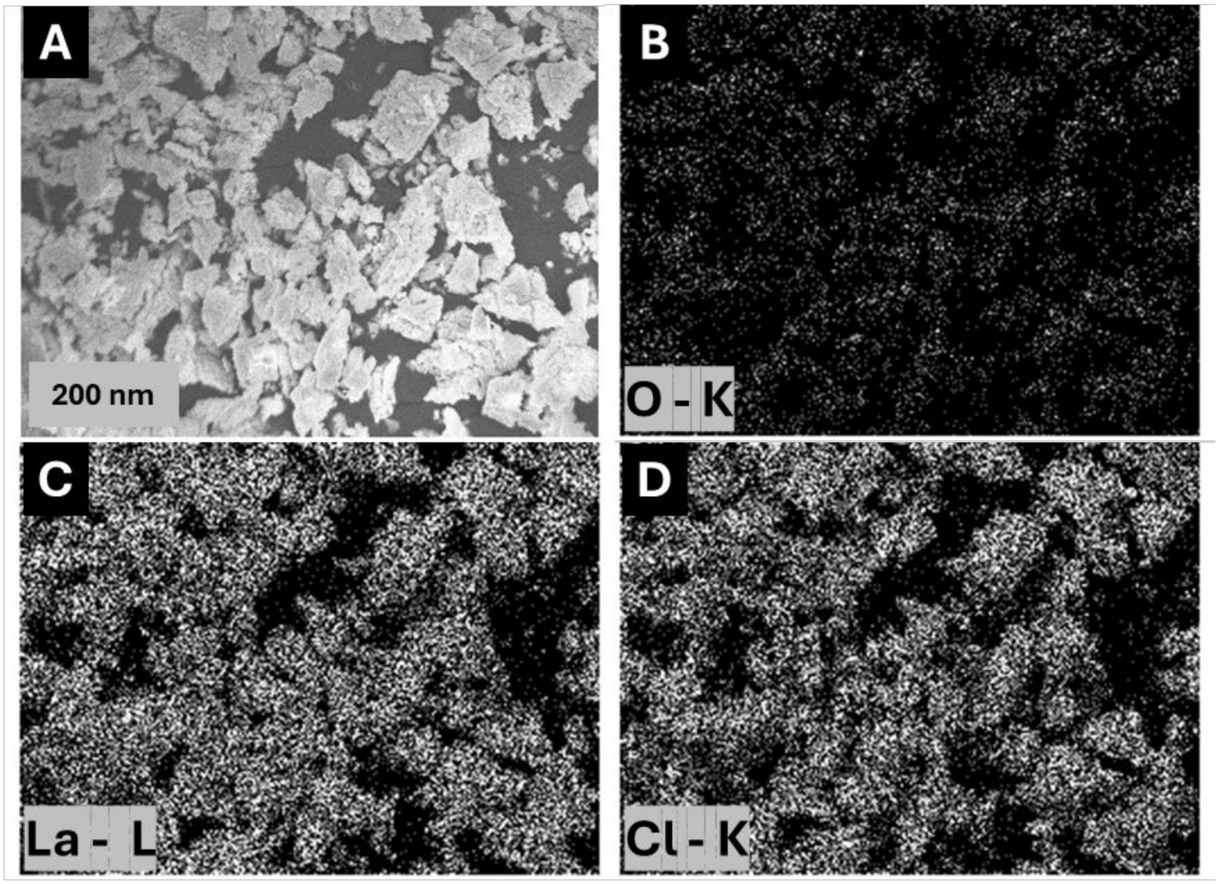

Figure S2: (A) SEM image of unalloyed LaOCl; Corresponding energy-dispersive X-ray spectroscopy (EDX) elemental maps showing the spatial distributions of (B) O, (C) La, and (D) Cl across the same region.

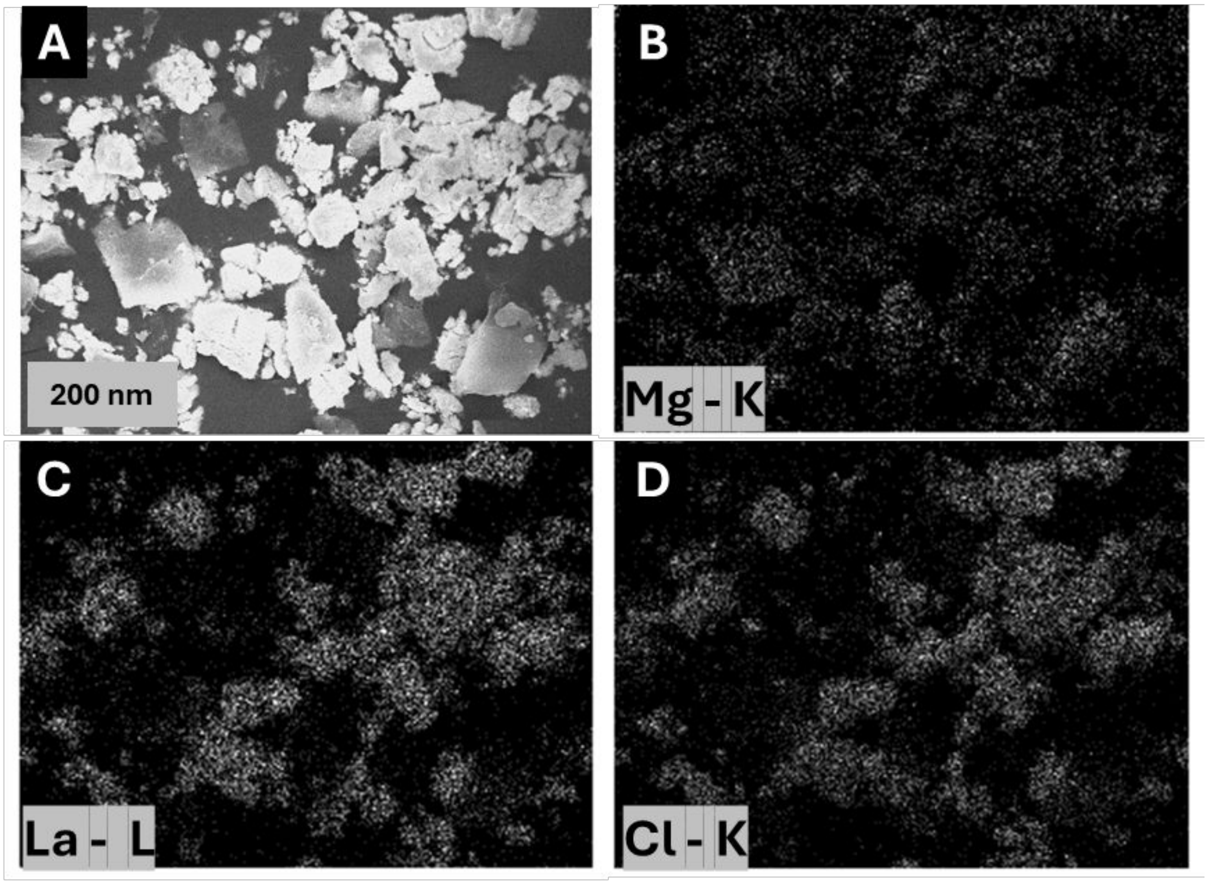

Figure S3: (A) SEM image of $La_{0.90}Mg_{0.08}OCl_{0.89}$; Corresponding EDX elemental maps showing the spatial distributions of (B) Mg, (C) La, and (D) Cl across the same region.

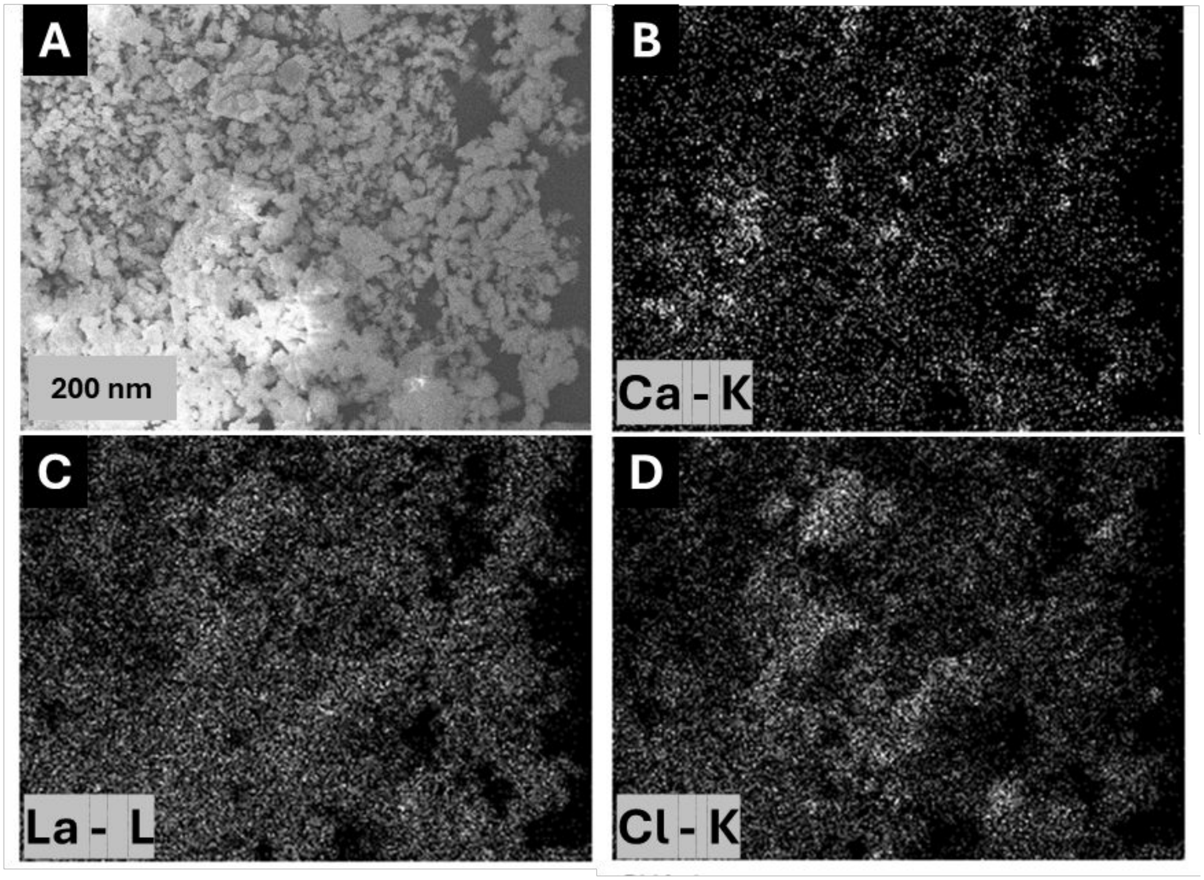

Figure S4:(A) SEM image of $La_{0.90}Ca_{0.08}OCl_{0.92}$; Corresponding EDX elemental maps showing the spatial distributions of (B) Ca, (C) La, and (D) Cl across the same region.

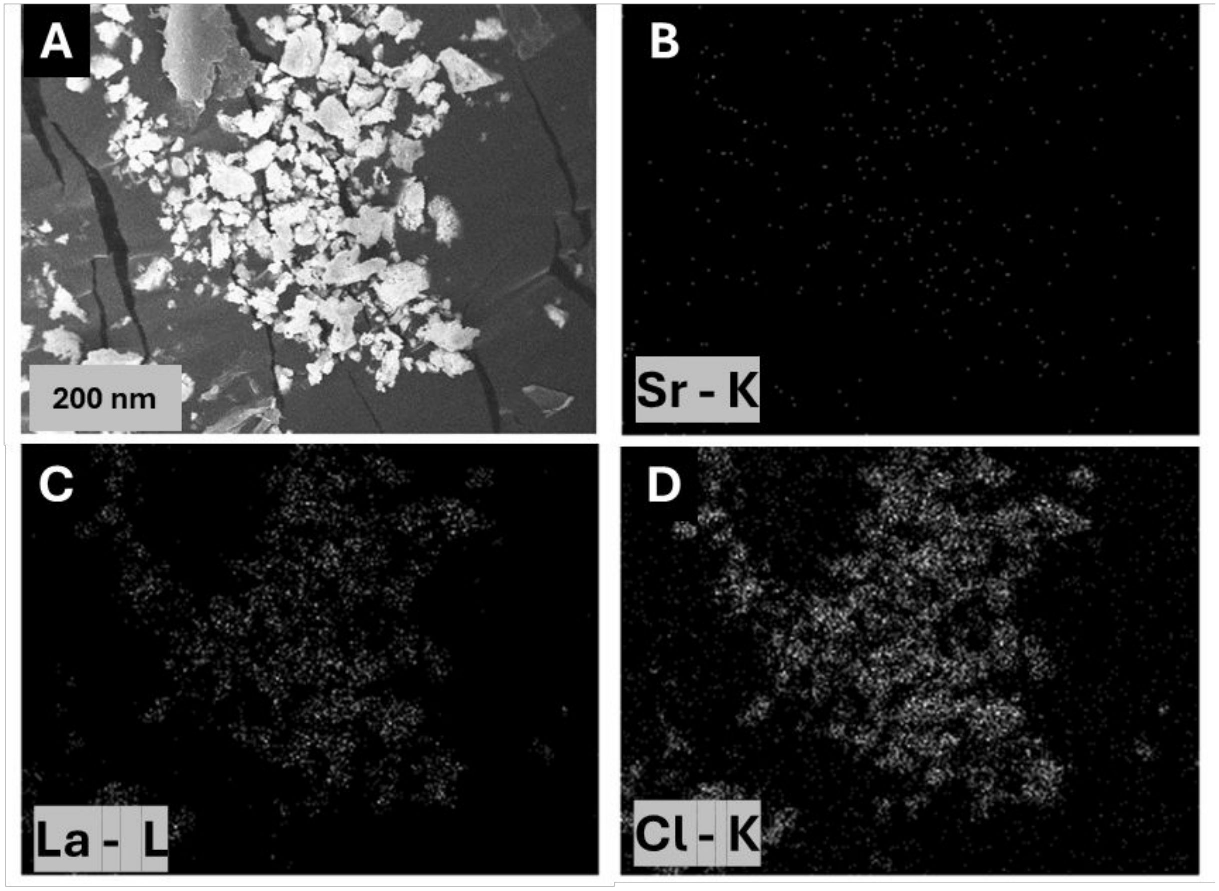

Figure S5: (A) SEM image of $La_{0.90}Sr_{0.09}OCl_{0.93}$; Corresponding EDX elemental maps showing the spatial distributions of (B) Sr, (C) La, and (D) Cl across the same region.

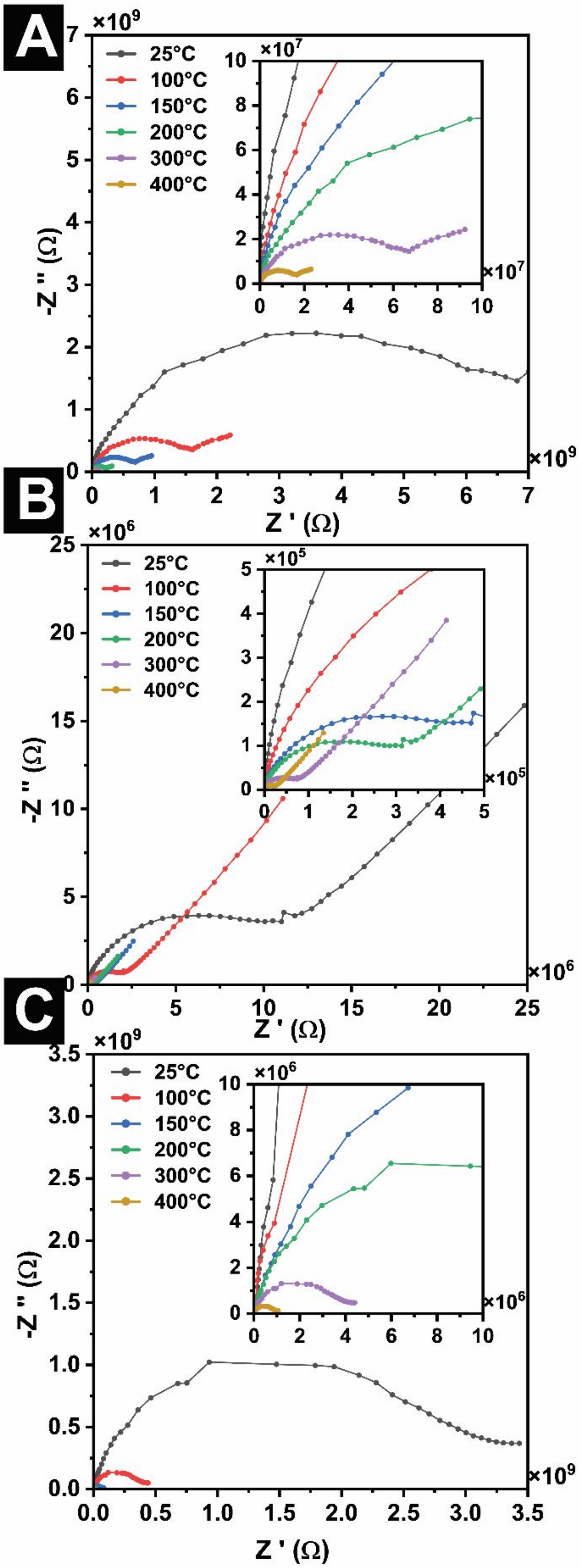

Figure S6: Nyquist plot for Mg-, Ca-, and Sr-alloyed LaOCl measured from 400 to 25 °C for (A) $La_{0.90}Mg_{0.08}OCl_{0.89}$; (B) $La_{0.90}Ca_{0.08}OCl_{0.92}$; and (C) $La_{0.90}Sr_{0.09}OCl_{0.93}$.

Table S3A: Equivalent-circuit (*R1 + (R2 || Q1) + (R3 || Q2) + W4*) fit parameters for LaOCl.

T (°C)	R1 (Ω)	R2 (Ω)	R3 (Ω)	*Q*1 ($S \cdot s^n$)	*n*1	*Q*2 ($S \cdot s^n$)	*n*2
25	2.40E+02	1.59E+08	4.24E+08	6.58E-09	0.84	3.68E-07	0.68
100	2.40E+02	2.04E+07	4.98E+07	9.36E-09	0.85	8.71E-07	0.69
150	2.35E+02	1.34E+07	3.09E+07	1.01E-08	0.85	1.13E-06	0.70
200	2.21E+02	7.16E+06	1.55E+07	1.12E-08	0.86	1.55E-06	0.71
300	1.95E+02	2.03E+06	3.94E+06	1.34E-08	0.87	2.86E-06	0.72
400	1.72E+02	7.53E+05	1.32E+06	1.50E-08	0.88	4.75E-06	0.74

Table S3B: Equivalent-circuit (*R1 + (R2 || Q1) + (R3 || Q2) + W4*) fit parameters for $La_{0.9}Mg_{0.08}OCl_{0.89}$.

T (°C)	R1 (Ω)	R2 (Ω)	R3 (Ω)	*Q*1 ($S \cdot s^n$)	*n*1	*Q*2 ($S \cdot s^n$)	*n*2
25	2.40E+02	1.69E+07	3.88E+07	7.47E-09	0.85	7.58E-07	0.69
100	2.40E+02	4.10E+06	8.67E+06	9.29E-09	0.86	1.42E-06	0.70
150	2.35E+02	1.82E+06	3.63E+06	1.04E-08	0.86	2.04E-06	0.71
200	2.21E+02	6.23E+05	1.18E+06	1.20E-08	0.87	3.08E-06	0.72
300	1.95E+02	2.01E+05	3.43E+05	1.35E-08	0.88	5.14E-06	0.74
400	1.72E+02	5.51E+04	8.48E+04	1.50E-08	0.89	8.59E-06	0.76

Table S3C: Equivalent-circuit (*R1 + (R2 || Q1) + (R3 || Q2) + W4*) fit parameters for $La_{0.9}Ca_{0.08}OCl_{0.92}$.

T (°C)	R1 (Ω)	R2 (Ω)	R3 (Ω)	*Q*1 ($S \cdot s^n$)	*n*1	*Q*2 ($S \cdot s^n$)	*n*2
25	2.40E+02	1.70E+05	2.02E+05	5.11E-09	0.88	2.55E-06	0.74
100	2.40E+02	7.38E+04	7.99E+04	5.42E-09	0.89	3.46E-06	0.75
150	2.35E+02	1.97E+04	2.01E+04	6.06E-09	0.89	5.01E-06	0.76
200	2.21E+02	5.80E+03	6.04E+03	6.59E-09	0.90	6.85E-06	0.77
300	1.95E+02	1.75E+03	2.05E+03	6.66E-09	0.91	9.14E-06	0.79
400	1.72E+02	4.70E+02	6.16E+02	6.63E-09	0.92	1.20E-05	0.81

Table S3D: Equivalent-circuit (*R1 + (R2 || Q1) + (R3 || Q2) + W4*) fit parameters for $La_{0.9}Sr_{0.09}OCl_{0.93}$.

T (°C)	R1 (Ω)	R2 (Ω)	R3 (Ω)	*Q*1 (S·s^n)	*n*1	*Q*2 (S·s^n)	*n*2
25	2.40E+02	7.06E+06	1.41E+07	7.64E-09	0.84	1.01E-06	0.70
100	2.40E+02	1.02E+06	1.85E+06	1.04E-08	0.85	2.13E-06	0.71
150	2.35E+02	1.99E+05	3.39E+05	1.31E-08	0.85	3.73E-06	0.72
200	2.21E+02	5.21E+04	8.35E+04	1.56E-08	0.86	5.87E-06	0.73
300	1.95E+02	1.32E+04	1.88E+04	1.79E-08	0.87	9.73E-06	0.75
400	1.72E+02	2.84E+03	3.60E+03	2.02E-08	0.88	1.60E-05	0.77

Table S3E: Equivalent-circuit (*R1 + (R2 || Q1) + (R3 || Q2) + W4*) fit parameters for $La_{0.9}Ca_{0.08}Mg_{0.01}OCl_{0.85}$.

T (°C)	R1 (Ω)	R2 (Ω)	R3 (Ω)	*Q*1 (S·s^n)	*n*1	*Q*2 (S·s^n)	*n*2
25	2.40E+02	3.75E+05	4.75E+05	5.61E-09	0.88	2.22E-06	0.73
100	2.40E+02	1.26E+05	1.45E+05	6.28E-09	0.89	3.34E-06	0.74
150	2.35E+02	5.24E+04	5.66E+04	6.82E-09	0.89	4.47E-06	0.75
200	2.21E+02	3.07E+04	3.11E+04	7.10E-09	0.89	5.42E-06	0.76
300	1.95E+02	4.71E+03	5.23E+03	8.10E-09	0.90	9.00E-06	0.78
400	1.72E+02	7.11E+02	8.88E+02	8.98E-09	0.91	1.40E-05	0.80

Table S3F: Equivalent-circuit (*R1 + (R2 || Q1) + (R3 || Q2) + W4*) fit parameters for $La_{0.9}Ca_{0.08}Mg_{0.04}OCl_{0.85}$.

T (°C)	R1 (Ω)	R2 (Ω)	R3 (Ω)	*Q*1 (S·s^n)	*n*1	*Q*2 (S·s^n)	*n*2
25	2.40E+02	9.15E+05	1.48E+06	7.31E-09	0.86	1.83E-06	0.71
100	2.40E+02	2.61E+05	3.87E+05	8.52E-09	0.87	3.00E-06	0.72
150	2.35E+02	1.13E+05	1.58E+05	9.31E-09	0.87	4.10E-06	0.73
200	2.21E+02	4.68E+04	6.16E+04	1.01E-08	0.88	5.59E-06	0.74
300	1.95E+02	1.58E+04	1.87E+04	1.08E-08	0.89	8.35E-06	0.76
400	1.72E+02	3.37E+03	3.57E+03	1.18E-08	0.90	1.33E-05	0.78

Table S3G: Equivalent-circuit (*R1 + (R2 || Q1) + (R3 || Q2) + W4*) fit parameters for $La_{0.9}Ca_{0.08}Mg_{0.05}OCl_{0.83}$.

T (°C)	R1 (Ω)	R2 (Ω)	R3 (Ω)	*Q*1 (S·s^n)	*n*1	*Q*2 (S·s^n)	*n*2
25	2.40E+02	4.35E+06	9.11E+06	6.80E-09	0.84	1.03E-06	0.69
100	2.40E+02	9.76E+05	1.85E+06	8.57E-09	0.85	1.94E-06	0.70
150	2.35E+02	4.16E+05	7.41E+05	9.66E-09	0.85	2.77E-06	0.71
200	2.21E+02	2.28E+05	3.82E+05	1.04E-08	0.86	3.64E-06	0.72
300	1.95E+02	8.74E+04	1.31E+05	1.14E-08	0.87	5.68E-06	0.74
400	1.72E+02	4.20E+04	5.61E+04	1.19E-08	0.88	8.14E-06	0.76

Table S3H: Equivalent-circuit (*R1 + (R2 || Q1) + (R3 || Q2) + W4*) fit parameters for $La_{0.9}Ca_{0.09}Sr_{0.01}OCl_{0.85}$.

T (°C)	R1 (Ω)	R2 (Ω)	R3 (Ω)	*Q*1 (S·s^n)	*n*1	*Q*2 (S·s^n)	*n*2
25	2.40E+02	1.20E+05	1.27E+05	5.41E-09	0.89	3.06E-06	0.75
100	2.40E+02	3.08E+04	3.18E+04	6.08E-09	0.90	4.62E-06	0.76
150	2.35E+02	9.51E+03	1.04E+04	6.66E-09	0.90	6.30E-06	0.77
200	2.21E+02	2.62E+03	3.05E+03	7.30E-09	0.90	8.66E-06	0.78
300	1.95E+02	7.02E+02	9.18E+02	7.62E-09	0.91	1.19E-05	0.79
400	1.72E+02	3.75E+02	5.50E+02	7.37E-09	0.92	1.38E-05	0.81

Table S3I: Equivalent-circuit (*R1 + (R2 || Q1) + (R3 || Q2) + W4*) fit parameters for $La_{0.9}Ca_{0.09}Sr_{0.03}OCl_{0.86}$.

T (°C)	R1 (Ω)	R2 (Ω)	R3 (Ω)	*Q*1 (S·s^n)	*n*1	*Q*2 (S·s^n)	*n*2
25	2.40E+02	3.78E+04	4.23E+04	5.68E-09	0.89	3.70E-06	0.75
100	2.40E+02	1.15E+04	1.41E+04	6.05E-09	0.90	5.19E-06	0.76
150	2.35E+02	3.56E+03	4.60E+03	6.48E-09	0.90	6.96E-06	0.77
200	2.21E+02	1.38E+03	1.89E+03	6.71E-09	0.91	8.70E-06	0.78
300	1.95E+02	3.70E+02	5.66E+02	6.67E-09	0.92	1.15E-05	0.80
400	1.72E+02	2.78E+02	4.76E+02	5.99E-09	0.93	1.21E-05	0.82

Table S3J: Equivalent-circuit (*R1 + (R2 || Q1) + (R3 || Q2) + W4*) fit parameters for $La_{0.9}Ca_{0.08}Sr_{0.05}OCl_{0.85}$.

T (°C)	R1 (Ω)	R2 (Ω)	R3 (Ω)	*Q*1 (S·s^n)	*n*1	*Q*2 (S·s^n)	*n*2
25	2.40E+02	1.84E+04	2.23E+04	5.05E-09	0.90	3.73E-06	0.76
100	2.40E+02	3.54E+03	4.68E+03	5.64E-09	0.91	5.69E-06	0.77
150	2.35E+02	6.89E+02	9.62E+02	6.31E-09	0.91	8.31E-06	0.78
200	2.21E+02	3.00E+02	4.43E+02	6.49E-09	0.91	9.97E-06	0.79
300	1.95E+02	2.01E+02	3.32E+02	6.11E-09	0.92	1.08E-05	0.80
400	1.72E+02	1.97E+02	2.79E+02	5.67E-09	0.93	1.14E-05	0.82

Table S4: Activation energy (slope/E_a) and Arrhenius prefactors (σ_0/y-intercept) derived from Arrhenius plot using $\log_{10}\sigma = b + m(1000/T)$ for LaOCl, $La_{0.90}Mg_{0.08}OCl_{0.89}$, $La_{0.90}Ca_{0.08}OCl_{0.92}$ and $La_{0.90}Sr_{0.09}OCl_{0.93}$.

Composition	Slope *m*	*y*-intercept *b*	E_a (eV)	Prefactor σ_0 (S/cm^2)	R^2
$La_{0.90}Ca_{0.08}OCl_{0.92}$	-1.41	-1.86	0.279	1.38E-2	0.958
$La_{0.90}Sr_{0.09}OCl_{0.93}$	-1.90	-1.93	0.377	1.18E-2	0.988
$La_{0.90}Mg_{0.08}OCl_{0.89}$	-1.38	-4.08	0.273	8.23E-5	0.975
LaOCl	-1.25	-5.38	0.249	4.21E-6	0.990

Table S4B: Activation energy (slope/E_a) and Arrhenius prefactors (σ_0/y-intercept) derived from Arrhenius plot using $\log_{10}\sigma = b + m(1000/T)$ for $La_{0.9}Ca_{0.08}Mg_{0.01}OCl_{0.85}$, $La_{0.9}Ca_{0.08}Mg_{0.04}OCl_{0.85}$, $La_{0.9}Ca_{0.08}Mg_{0.05}OCl_{0.83}$, $La_{0.9}Ca_{0.09}Sr_{0.01}OCl_{0.85}$, $La_{0.9}Ca_{0.09}Sr_{0.03}OCl_{0.86}$, and $La_{0.9}Ca_{0.08}Sr_{0.05}OCl_{0.85}$.

Composition	Slope *m*	*y*-intercept *b*	E_a (eV)	Prefactor σ_0 (S/cm^2)	R^2
$La_{0.9}Ca_{0.09}Sr_{0.01}OCl_{0.85}$	-1.123	-1.930	0.223	1.17E-2	0.938
$La_{0.9}Ca_{0.09}Sr_{0.03}OCl_{0.86}$	-1.193	-2.219	0.237	6.04E-3	0.976
$La_{0.9}Ca_{0.08}Sr_{0.05}OCl_{0.85}$	-1.396	-2.072	0.277	8.48E-3	0.978
$La_{0.9}Ca_{0.08}Mg_{0.01}OCl_{0.85}$	-1.419	-2.693	0.282	2.03E-3	0.920
$La_{0.9}Ca_{0.08}Mg_{0.04}OCl_{0.85}$	-1.340	-3.324	0.266	4.75E-4	0.959
$La_{0.9}Ca_{0.08}Mg_{0.05}OCl_{0.83}$	-1.119	-4.634	0.222	2.32E-5	0.997

Table S5: Matrix densities and theoretical crystallographic densities of unalloyed and alloyed LaOCl measured by helium pycnometry.

Sample	Matrix Density (g/mL)	Theoretical Crystallographic Density (g/mL)	Difference (Δ%)
LaOCl	4.689	5.118	-8.4
$La_{0.90}Mg_{0.08}OCl_{0.89}$	4.740	4.984	-4.9
$La_{0.90}Ca_{0.08}OCl_{0.92}$	5.090	5.035	1.1
$La_{0.90}Sr_{0.09}OCl_{0.93}$	4.713	5.137	-8.3
$La_{0.9}Ca_{0.08}Mg_{0.01}OCl_{0.85}$	5.309	4.976	6.7
$La_{0.9}Ca_{0.08}Mg_{0.04}OCl_{0.85}$	5.370	4.992	7.6
$La_{0.9}Ca_{0.08}Mg_{0.05}OCl_{0.83}$	5.410	4.973	8.8
$La_{0.9}Ca_{0.09}Sr_{0.01}OCl_{0.85}$	5.434	5.001	8.7
$La_{0.9}Ca_{0.09}Sr_{0.03}OCl_{0.86}$	5.090	5.044	0.9
$La_{0.9}Ca_{0.08}Sr_{0.05}OCl_{0.85}$	5.280	5.058	4.4

Table S6: Mole ratio of all precursors used, expected, and actual product composition of Dy, Mg, Ca and Sr co-alloyed products.

Precursors (mole ratio)				Expected product composition	Actual product composition
yDy_2O_3	$(1-x-y)$ La_2O_3	$2x$ $(COO)_2Ca$	$(2-2x$ NH_4Cl	$La_{1-x-y}Dy_yCa_xOCl_{1-x}$	$La_{1-x-y}Dy_yCa_xOCl_{1-x}$
0.01	0.99	0	2	$La_{0.99}Dy_{0.012}OCl_{1.07}$	$La_{0.99}Dy_{0.012}OCl_{1.07}$
yDy_2O_3	$(1-x-y)$ La_2O_3	$2x$ $(COO)_2Ca$	$(2-2x$ NH_4Cl	$La_{1-x-y}Dy_yCa_xOCl_{1-x}$	$La_{1-x-y}Dy_yCa_xOCl_{1-x}$
0.01	0.89	0.2	1.8	$La_{0.9}Dy_{0.01}Mg_{0.1}OCl_{0.9}$	$La_{0.9}Dy_{0.012}Mg_{0.14}OCl_{0.92}$
yDy_2O_3	$(1-x-y)$ La_2O_3	$2x$ $(COO)_2Ca$	$(2-2x$ NH_4Cl	$La_{1-x-y}Dy_yCa_xOCl_{1-x}$	$La_{1-x-y}Dy_yCa_xOCl_{1-x}$
0.01	0.89	0.2	1.8	$La_{0.9}Dy_{0.01}Ca_{0.1}OCl_{0.9}$	$La_{0.9}Dy_{0.013}Ca_{0.1}OCl_{0.94}$
yDy_2O_3	$(1-x-y)$ La_2O_3	$2x$ $(COO)_2Ca$	$(2-2x$ NH_4Cl	$La_{1-x-y}Dy_yCa_xOCl_{1-x}$	$La_{1-x-y}Dy_yCa_xOCl_{1-x}$
0.01	0.89	0.2	1.8	$La_{0.9}Dy_{0.01}Ca_{0.1}OCl_{0.9}$	$La_{0.9}Dy_{0.02}Sr_{0.11}OCl_{0.93}$

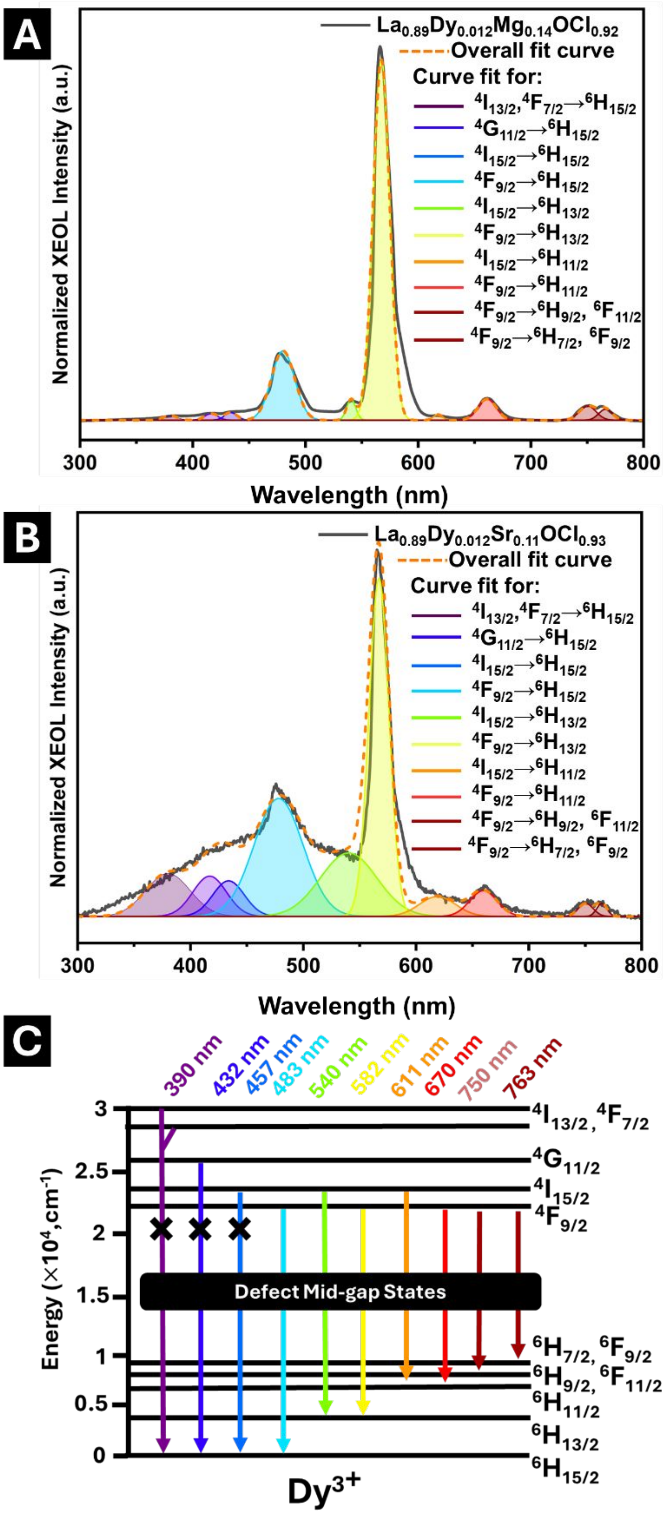

Figure S7: FWHM curve fit of XEOL spectra for (A) $La_{0.9}Dy_{0.012}Mg_{0.14}OCl_{0.92}$; (B) $La_{0.9}Dy_{0.02}Sr_{0.11}OCl_{0.93}$; (C) Dieke diagram illustrating sensitized emissions.

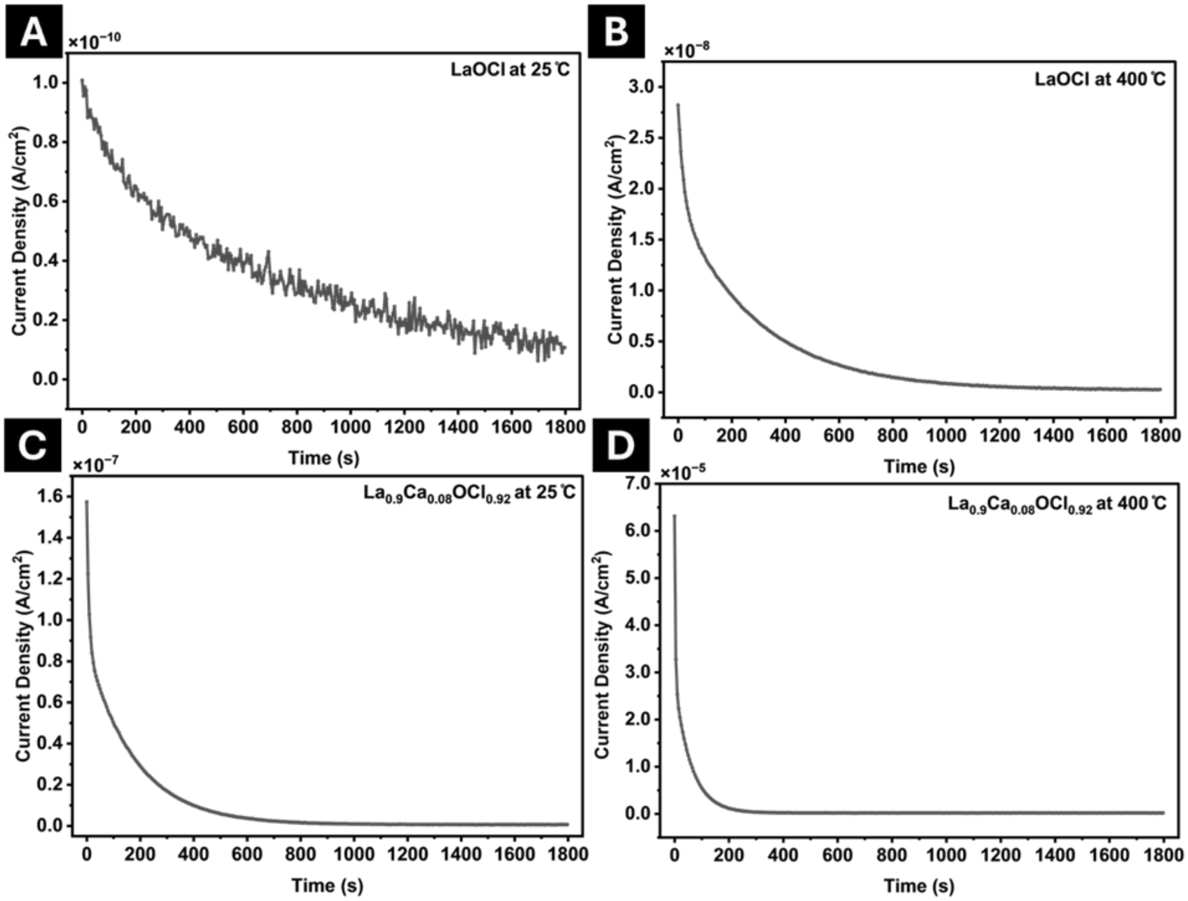

Figure S8: DC polarization measurements on (A) unalloyed LaOCl at 25 °C; (B) unalloyed LaOCl at 400 °C; (C) $La_{0.9}Ca_{0.08}OCl_{0.92}$ at 25 °C; and (D) $La_{0.9}Ca_{0.08}OCl_{0.92}$ at 400 °C.

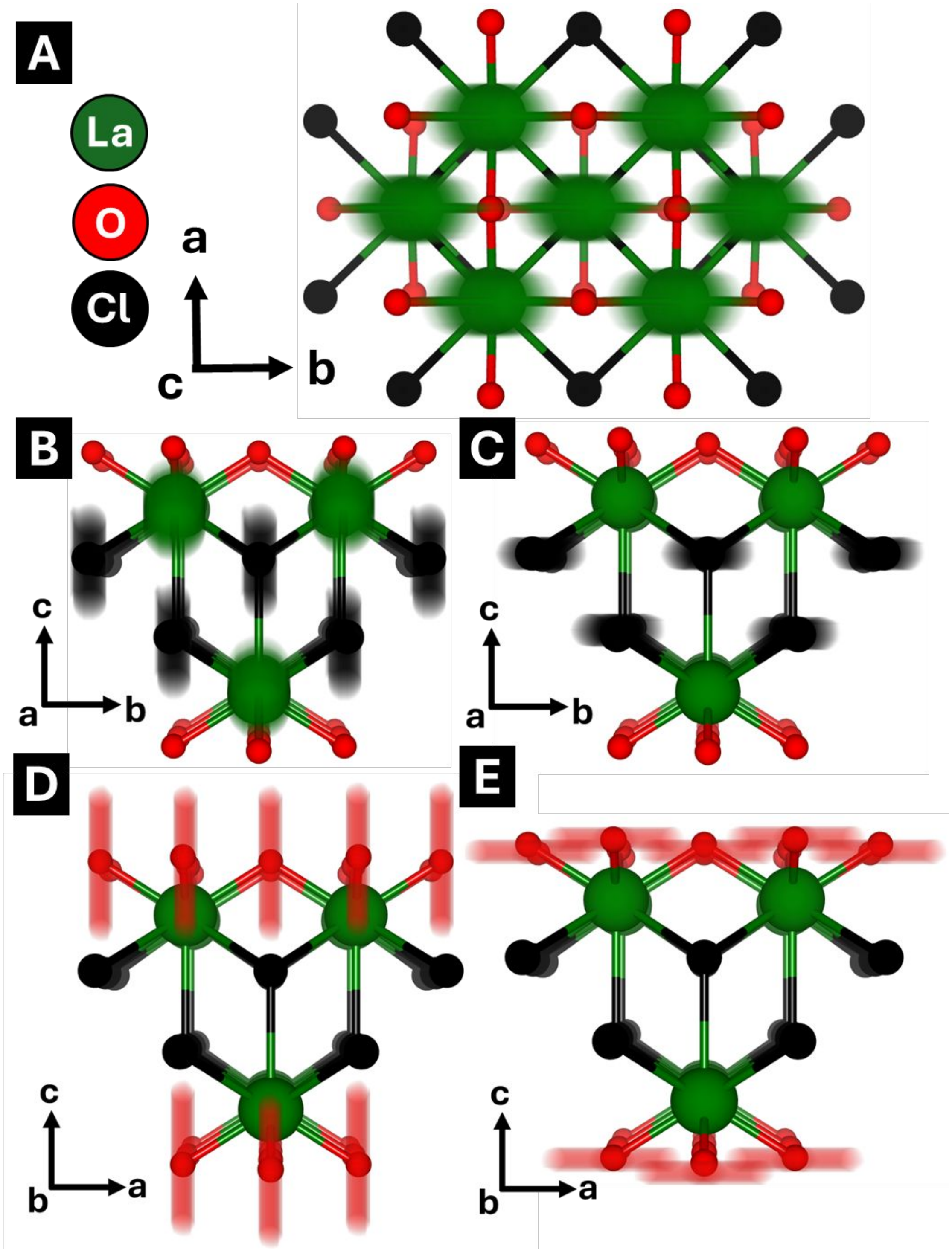

Figure S9: Visualization of Γ-point lattice vibrational modes in LaOCl corresponding to the Raman active bands. (A)Representative displaces patterns for La-Cl – A_{1g} breathing-type vibration within the *ab* plane; (B) La/Cl-Cl – A_{1g} mode involving predominantly apical Cl motion along the *c*-axis; (C) Cl-Cl – E_g shear-like mode with in-plane halide displacements in the *ab*-plane; (D) higher intensity layer-breathing along O-La – E_g mode *c*-axis; and (E) higher intensity in-plane anti-phase shear O-La – B_{1g} mode along *a*-axis.

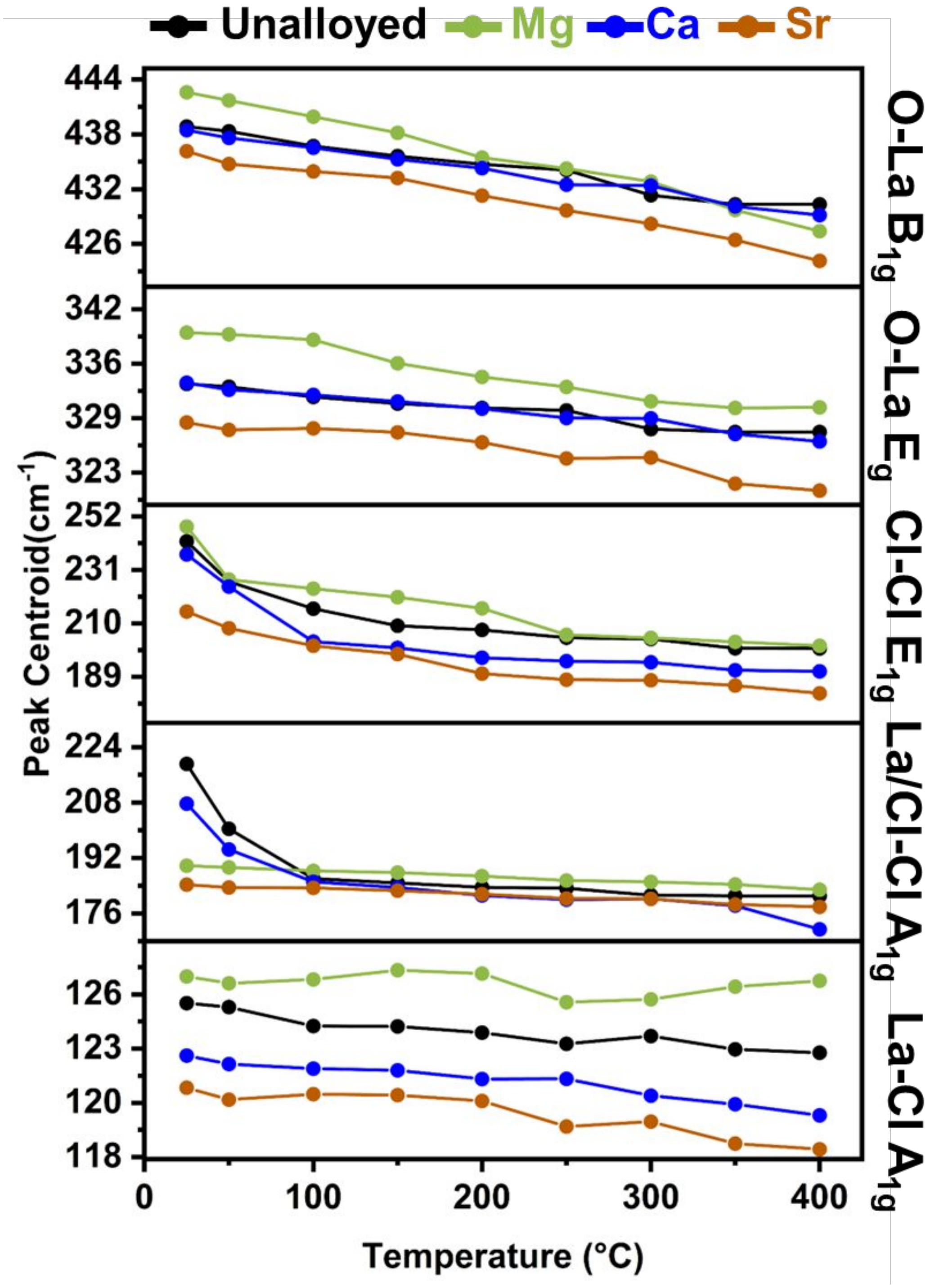

Figure S10: Temperature dependence of Raman peak positions for unalloyed and aliovalently alloyed LaOCl. Centroids of five representative Raman modes are plotted as a function of temperature (25–400 °C) for unalloyed LaOCl (black) and Mg-, Ca-, and Sr-alloyed LaOCl (green, blue, and orange, respectively).

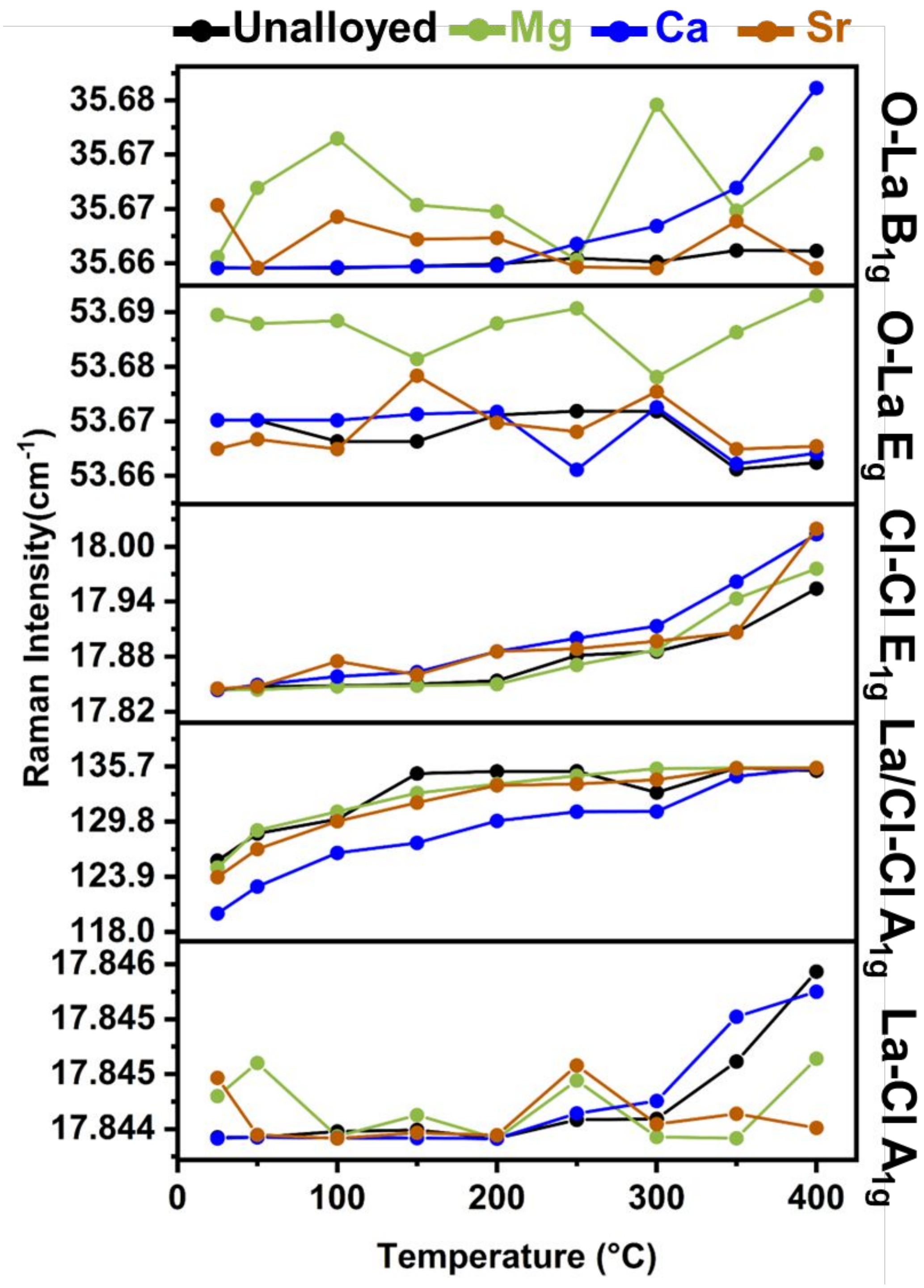

Figure S11: Temperature dependence of Raman band widths for unalloyed and aliovalently alloyed LaOCl. Full-width-at-half-maximum (FWHM) of the same five Raman bands is plotted as a function of temperature (25–400 °C) for unalloyed LaOCl (black) and Mg-, Ca-, and Sr-alloyed LaOCl (green, blue, and orange, respectively).

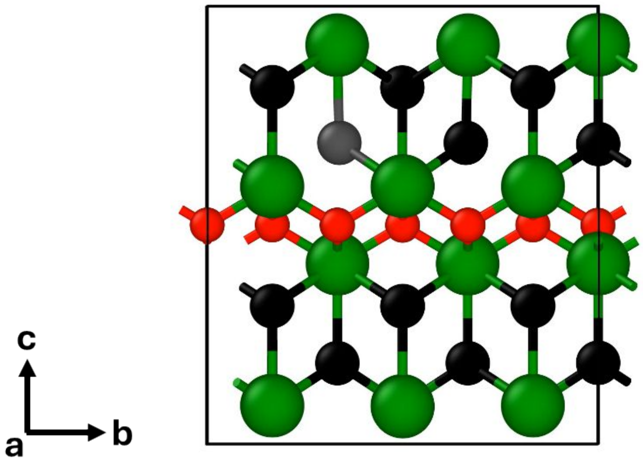

Video S1: Cl-ion migration pathway derived from cAIMD simulations of unalloyed LaOCl.

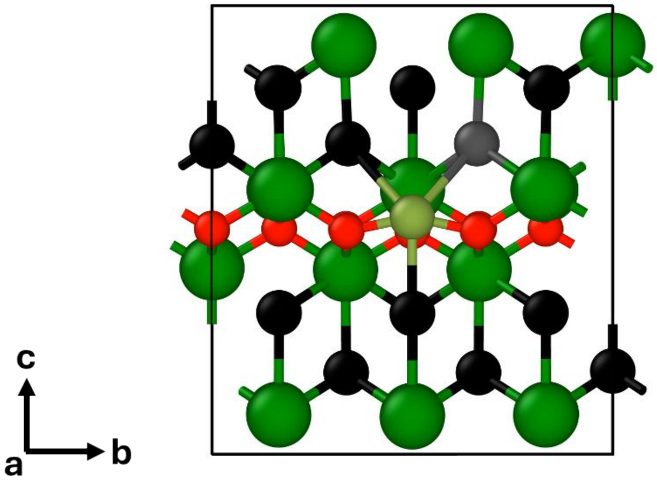

Animation S2: Cl-ion migration pathway derived from cAIMD simulations of $La_{0.92}Mg_{0.08}OCl_{0.92}$.

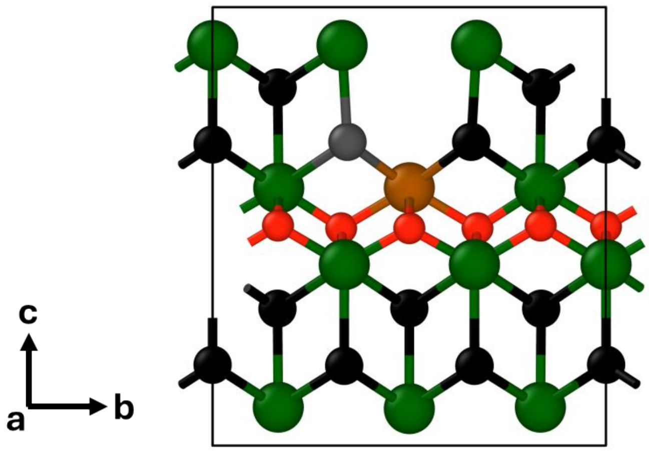

Animation S3: Cl-ion migration pathway derived from cAIMD of $La_{0.92}Sr_{0.08}OCl_{0.92}$.

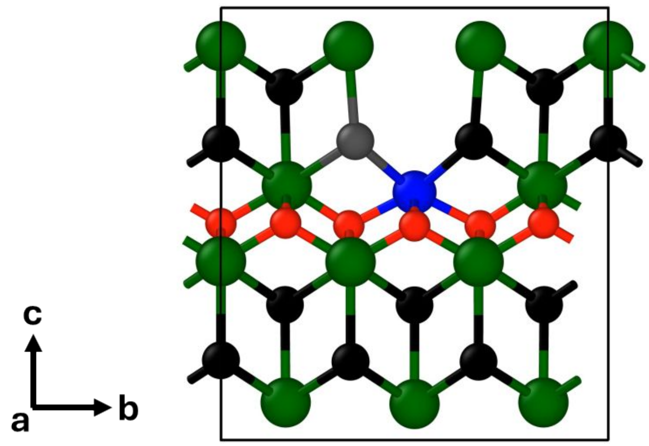

Animation S4: Cl-ion migration pathway derived from cAIMD of $La_{0.92}Ca_{0.08}OCl_{0.92}$.

Table S7: Mole ratio of all precursors used, expected, and actual product composition of Mg, Ca and Sr co-alloyed compounds.

Precursors (Mole Ratio)				Expected product composition	Actual product composition
$(1-x-y$ La_2O_3	$2x(COO)_2$ Ca	$2y(COO)_2$ Mg	$(2-2x-y$ NH_4Cl	$La_{1-x-y}Mg_y$ Ca_xOCl_{1-x-y}	$La_{1-x-y}Mg_y$ Ca_xOCl_{1-x-y}
0.85	0.2	0.1	1.7	$La_{0.9}Mg_{0.05}Ca_{0.1}OCl_{0.85}$	$La_{0.9}Mg_{0.05}Ca_{0.08}OCl_{0.83}$
0.87	0.2	0.06	1.74	$La_{0.9}Mg_{0.03}Ca_{0.1}OCl_{0.87}$	$La_{0.9}Mg_{0.04}Ca_{0.08}OCl_{0.85}$
0.89	0.2	0.02	1.78	$La_{0.9}Mg_{0.01}Ca_{0.1}OCl_{0.89}$	$La_{0.9}Mg_{0.01}Ca_{0.08}OCl_{0.85}$
$(1-x-y$ La_2O_3	$2x(COO)_2$ Ca	$2y(COO)_2$ Sr	$(2-2x-y$ NH_4Cl	$La_{1-x-y}Sr_y$ Ca_xOCl_{1-x-y}	$La_{1-x-y}Sr_y$ Ca_xOCl_{1-x-y}
0.85	0.2	0.1	1.7	$La_{0.9}Sr_{0.05}Ca_{0.1}OCl_{0.85}$	$La_{0.9}Sr_{0.05}Ca_{0.08}OCl_{0.85}$
0.87	0.2	0.06	1.74	$La_{0.9}Sr_{0.03}Ca_{0.1}OCl_{0.87}$	$La_{0.9}Sr_{0.03}Ca_{0.09}OCl_{0.86}$
0.89	0.2	0.02	1.78	$La_{0.9}Sr_{0.01}Ca_{0.1}OCl_{0.89}$	$La_{0.9}Sr_{0.01}Ca_{0.09}OCl_{0.85}$

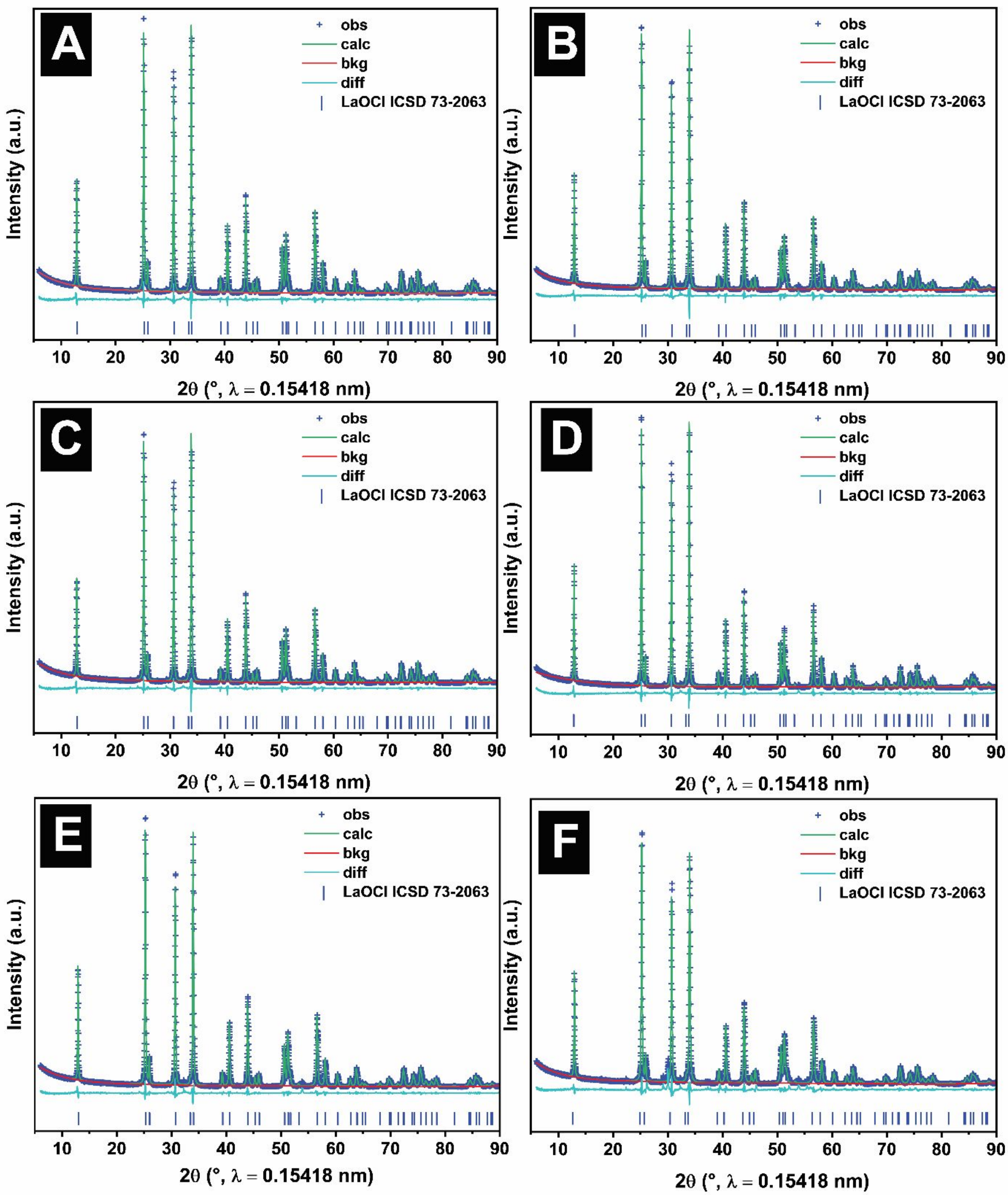

Figure S11: Rietveld refinement of powder XRD patterns for Ca-centered co-alloyed LaOCl compositions of (A) $La_{0.9}Ca_{0.08}Mg_{0.01}OCl_{0.85}$; (B) $La_{0.9}Ca_{0.08}Mg_{0.04}OCl_{0.85}$; (C) $La_{0.9}Ca_{0.08}Mg_{0.05}OCl_{0.83}$; (D) $La_{0.9}Ca_{0.09}Sr_{0.01}OCl_{0.85}$; (E) $La_{0.9}Ca_{0.09}Sr_{0.03}OCl_{0.86}$; and (F) $La_{0.9}Ca_{0.08}Sr_{0.05}OCl_{0.85}$.

Table S8A. Refined lattice parameters, atomic positions and thermal parameters for $La_{0.9}Ca_{0.08}Mg_{0.01}OCl_{0.85}$.

$La_{0.9}Ca_{0.08}Mg_{0.01}OCl_{0.85}$						
wR	10.09%	R	7.76%	χ^2	2.90	
2θ range	6 - 90°	Radiation	Cu Kα 1.5406 Å	Temp	295 K	
Formula	$La_{1-x-y}Ca_xMg_yOCl_{1-x-y}$	Z	2	V (Å³)	116.53	
a=b (Å)	4.1169(6)	*c (Å)*	6.8754(7)	S.G.	*P4/nmm*	
Atom	*x*	*y*	*z*	frac.	Wyckoff	Uiso
La	0.0015(2)	0.5045(7)	0.1763(7)	0.90	2c	0.0049(4)
Ca	0.0018(1)	0.4985(5)	0.1751(4)	0.08	2c	0.0068(4)
Mg	0.0012(6)	0.5024(2)	0.1774(6)	0.01	2c	0.0058(2)
O	0.9990(4)	0.9997(4)	0.0009(8)	1	2a	0.0137(6)
Cl	0.0002(7)	0.5007(3)	0.6325(8)	0.85	2c	0.0137(3)

Table S8B. Refined lattice parameters, atomic positions and thermal parameters for $La_{0.9}Ca_{0.08}Mg_{0.04}OCl_{0.85}$.

$La_{0.9}Ca_{0.08}Mg_{0.04}OCl_{0.85}$						
wR	10.21%	R	7.88%	χ^2	2.54	
2θ range	6 - 90°	Radiation	Cu Kα 1.5406 Å	Temp	295 K	
Formula	$La_{1-x-y}Ca_xMg_yOCl_{1-x-y}$	Z	2	V (Å³)	116.631	
a=b (Å)	4.1177(9)	*c (Å)*	6.8787(6)	S.G.	*P4/nmm*	
Atom	*x*	*y*	*z*	frac.	Wyckoff	Uiso
La	0.0005(6)	0.5036(3)	0.1728(7)	0.90	2c	0.0035(8)
Ca	0.0010(5)	0.5009(4)	0.1724(6)	0.08	2c	0.0044(3)
Mg	0.0004(1)	0.5072(7)	0.1765(6)	0.04	2c	0.0064(3)
O	0.9984(2)	0.0006(5)	0.0009(6)	1	2a	0.0149(7)
Cl	0.0006(6)	0.4998(6)	0.6318(8)	0.85	2c	0.0128(2)

Table S8C. Refined lattice parameters, atomic positions and thermal parameters for $La_{0.9}Ca_{0.08}Mg_{0.05}OCl_{0.83}$.

$La_{0.9}Ca_{0.08}Mg_{0.05}OCl_{0.83}$						
wR	10.62%	R	8.04%	χ^2	2.85	
2θ range	6 - 90°	Radiation	Cu Kα 1.5406 Å	Temp	295 K	
Formula	$La_{1-x-y}Ca_xMg_yOCl_{1-x-y}$	Z	2	V (Å³)	116.77	
a=b (Å)	4.1190(9)	*c (Å)*	6.8825(6)	S.G.	*P4/nmm*	
Atom	*x*	*y*	*z*	frac	Wyckoff	Uiso
La	0.0021(5)	0.5044(4)	0.1764(5)	0.90	2c	0.0036(8)
Ca	0.0013(7)	0.5009(3)	0.1741(7)	0.08	2c	0.0055(3)
Mg	0.9996(2)	0.5034(1)	0.1741(2)	0.05	2c	0.0065(2)
O	0.9997(6)	0.9992(2)	0.0021(9)	1	2a	0.0132(7)
Cl	0.0019(6)	0.4997(1)	0.6314(9)	0.83	2c	0.0118(7)

Table S8D. Refined lattice parameters, atomic positions and thermal parameters for $La_{0.9}Ca_{0.09}Sr_{0.01}OCl_{0.85}$.

$La_{0.9}Ca_{0.09}Sr_{0.01}OCl_{0.85}$						
wR	10.97%	R	7.81%	χ^2	2.89	
2θ range	6 - 90°	Radiation	Cu Kα 1.5406 Å	Temp	295 K	
Formula	$La_{1-x-y}Ca_xSr_yOCl_{1-x-y}$	Z	2	V (Å³)	116.634	
a=b (Å)	4.1185(7)	*c (Å)*	6.8762(9)	S.G.	*P4/nmm*	
Atom	*x*	*y*	*z*	frac	Wyckoff	Uiso
La	0.0018(2)	0.5043(4)	0.1744(6)	0.90	2c	0.0043(8)
Ca	0.0035(4)	0.5033(5)	0.1732(2)	0.09	2c	0.0050(3)
Sr	0.0025(7)	0.5006(6)	0.1717(4)	0.01	2c	0.0057(3)
O	0.9987(5)	0.9999(7)	0.0012(5)	1	2a	0.0128(6)
Cl	0.0005(2)	0.4998(4)	0.6330(6)	0.85	2c	0.0108(7)

Table S8E. Refined lattice parameters, atomic positions and thermal parameters for $La_{0.9}Ca_{0.09}Sr_{0.03}OCl_{0.86}$.

$La_{0.9}Ca_{0.09}Sr_{0.03}OCl_{0.86}$						
wR	10.48%	R	7.51%	χ^2	2.31	
2θ range	6 - 90°	Radiation	Cu Kα 1.5406 Å	Temp	295 K	
Formula	$La_{1-x-y}Ca_xSr_yOCl_{1-x-y}$	Z	2	V (Å³)	117.024	
a=b (Å)	4.1235(8)	*c (Å)*	6.8828(6)	S.G.	*P4/nmm*	
Atom	*x*	*y*	*z*	frac	Wyckoff	Uiso
La	0.0005(5)	0.5027(7)	0.1767(4)	0.90	2c	0.0043(7)
Ca	0.0007(2)	0.5008(6)	0.1744(4)	0.09	2c	0.0070(6)
Sr	0.0037(4)	0.4985(7)	0.1706(7)	0.03	2c	0.0056(4)
O	0.9990(3)	0.0016(3)	0.0024(5)	1	2a	0.0150(4)
Cl	0.0015(1)	0.4980(7)	0.6332(6)	0.86	2c	0.0113(6)

Table S8F. Refined lattice parameters, atomic positions and thermal parameters for $La_{0.9}Ca_{0.08}Sr_{0.05}OCl_{0.85}$.

$La_{0.9}Ca_{0.08}Sr_{0.05}OCl_{0.85}$						
wR	10.83%	R	8.10%	χ^2	2.71	
2θ range	6 - 90°	Radiation	Cu Kα 1.5406 Å	Temp	295 K	
Formula	$La_{1-x-y}Ca_xSr_yOCl_{1-x-y}$	Z	2	V (Å³)	117.367	
a=b (Å)	4.1276(6)	*c (Å)*	6.8889(7)	S.G.	*P4/nmm*	
Atom	*x*	*y*	*z*	frac	Wyckoff	Uiso
La	0.0009(4)	0.5015(2)	0.1750(6)	0.90	2c	0.0042(8)
Ca	0.0026(3)	0.5001(3)	0.1729(5)	0.08	2c	0.0045(6)
Sr	0.0032(6)	0.4987(3)	0.1743(9)	0.05	2c	0.0079(2)
O	0.9997(5)	0.0009(2)	0.0026(2)	1	2a	0.0136(3)
Cl	0.0006(6)	0.5006(4)	0.6322(8)	0.85	2c	0.0117(6)

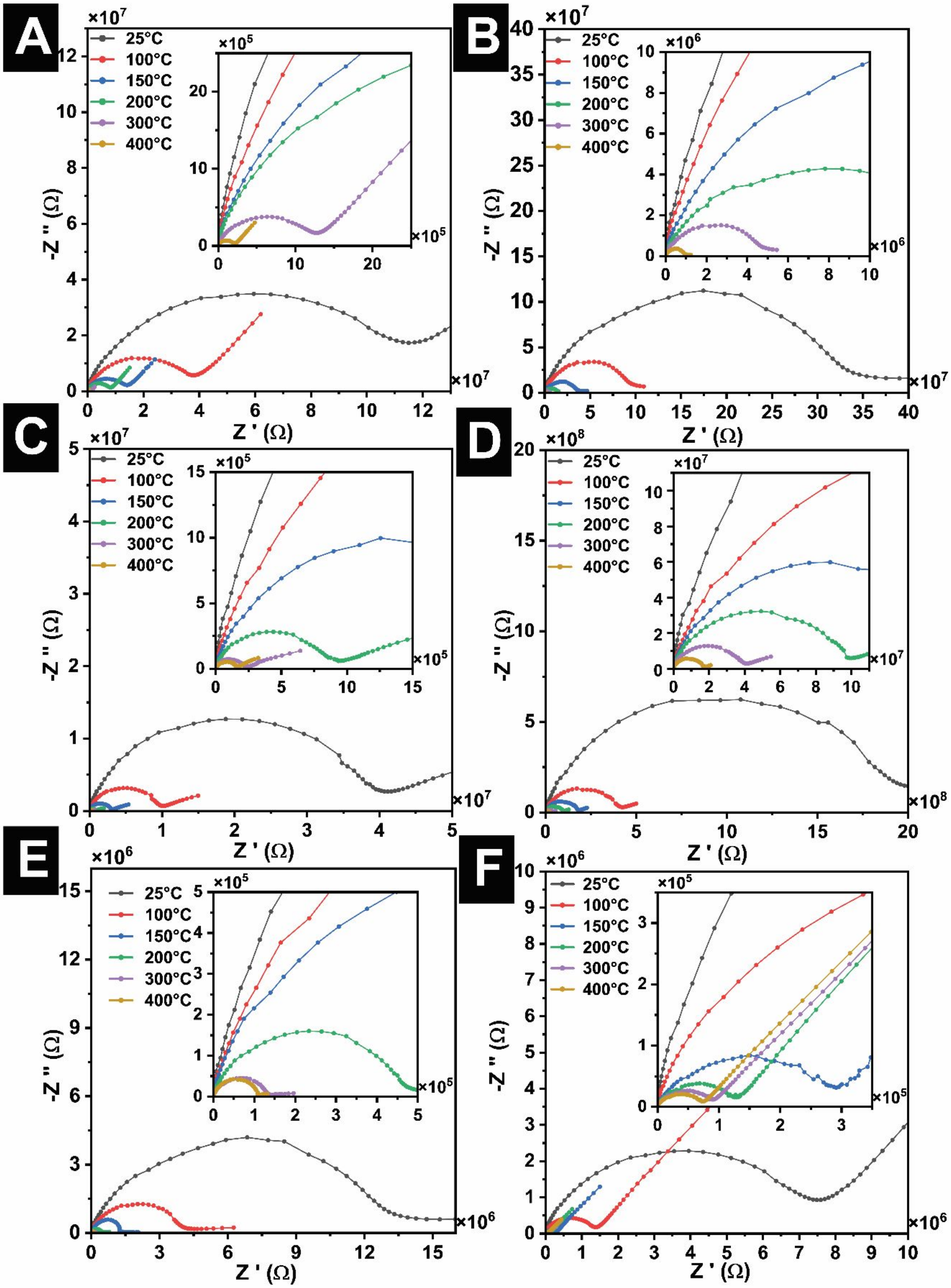

Figure S13: Nyquist plot for co-alloyed LaOCl from 400 to 25 °C for (A) $La_{0.9}Ca_{0.08}Mg_{0.01}OCl_{0.85}$; (B) $La_{0.9}Ca_{0.08}Mg_{0.04}OCl_{0.85}$; (C) $La_{0.9}Ca_{0.08}Mg_{0.05}OCl_{0.83}$; (D) $La_{0.9}Ca_{0.09}Sr_{0.01}OCl_{0.85}$; (E) $La_{0.9}Ca_{0.09}Sr_{0.03}OCl_{0.86}$; and (F) $La_{0.9}Ca_{0.08}Sr_{0.05}OCl_{0.85}$.
